# Environmental determinants of West Nile virus vector abundance at the wildlife–livestock interface

**DOI:** 10.1111/mve.12774

**Published:** 2024-11-05

**Authors:** Laia Casades‐Martí, Alfonso Peralbo‐Moreno, Sarah Delacour‐Estrella, Francisco Ruiz‐Fons

**Affiliations:** ^1^ Grupo Sanidad y Biotecnología (SaBio) Instituto de Investigación en Recursos Cinegéticos (IREC), CSIC–UCLM–JCCM Ciudad Real Spain; ^2^ Unizar, Departamento de Patologia Animal, Facultad de Veterinaria Universidad de Zaragoza Zaragoza Spain; ^3^ CIBERINFEC, Centro de Investigación Biomédica en Red de Enfermedades Infecciosas Instituto de Salud Carlos III Madrid Spain

**Keywords:** *Culex pipiens*, ecology, *Flavivirus*, mosquito, zoonosis

## Abstract

The diversity and abundance of vectors are essential parameters in the transmission dynamics of West Nile virus (WNV) between its avian reservoirs and clinically susceptible mammalian species. Knowing the determinants of vector abundance could be thus useful in preventing West Nile fever (WNF) cases and associated socio‐economic impact. We designed a survey at the wildlife–livestock interface to test the hypothesis that variations in environmental favourability between anthropized and wild scenarios modulate WNV vector abundance and transmission risk. In a continental Mediterranean region where WNF has recently emerged, we selected nine sampling sites and allocated three areas to every site with a decreasing gradient of wildlife–livestock interaction: A_1_—a horse farm where interaction is maximal; A_2_—a zone of intermediate interaction 500–1000 m from the farm; and A_3_—an entirely wild zone of low interaction 1–5 km from the farm. At a fortnightly frequency, we estimated mosquito abundance at each of the 27 study sites in May–December 2018 and April–July 2019. We estimated bird and mammal abundance, collected meteorological information and characterised mosquito habitat at the site scale. Thereafter, we studied the determinants of *Culex* spp., *Culex pipiens* sensu lato (s.l.) Linnaeus, 1758 (Diptera: Culicidae) and *Culex theileri*  Theobald, 1903 abundance by constructing negative binomial generalised linear mixed models. We identified 20 mosquito species, with a notable predominance of *Culex* spp. and, particularly, of *Cx. pipiens* s.l. We found differences in the spatiotemporal distribution of *Culex* spp. abundance and confirmed our hypothesis by finding important effects of local environmental variations in abundance. The accumulated rainfall in fortnights 4–14 and the mean temperature of the two fortnights before sampling were positively and statistically significantly associated with the abundance of *Cx. pipiens* s.l. (*Z* = 13.09, *p* < 0.001, and *Z* = 9.91, *p* < 0. 001, respectively) and *Culex* spp. (*Z* = 13.35, *p* < 0.001, and *Z* = 6.99, *p* < 0.001, respectively), while the mean temperature of the two previous fortnights was a positive statistically significant predictor (*Z* = 14.69, *p* < 0.001) of the abundance of *Cx. theileri*. The farm environment was the most conducive predictor to hosting *Culex* spp. compared with wild settings. Our results indicate that continental Mediterranean environments are favourable for WNV circulation and maintenance, especially the environment of anthropized rural settings such as farms. These results will have an impact on the spatiotemporal risk prediction of WNF emergence in continental Mediterranean environments.

## Graphical abstract

- Mosquito communities in southern inland Iberia are dominated by highly competent vectors of West Nile virus.
- The coexistence of *Culex pipiens* and *Culex theileri* in southern inland Iberia indicates that both species should be involved in West Nile virus transmission.
- West Nile virus vector abundance is highly modulated by rainfall and temperature regimes in continental areas of the southern Iberian Peninsula.

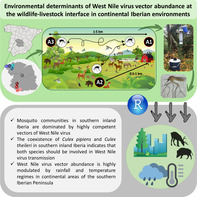

## RESUMEN

La diversidad y abundancia de vectores son parámetros esenciales en la dinámica de transmisión del virus del Nilo Occidental (VNO) entre sus reservorios aviares y las especies de mamíferos clínicamente susceptibles. Conocer los determinantes de la abundancia de vectores es, por tanto, primordial para prevenir los casos de fiebre del Nilo Occidental (FNO) y el impacto socioeconómico asociado. Diseñamos un estudio en la interfaz entre fauna silvestre y ganadería para comprobar la hipótesis de que las variaciones en la favorabilidad ambiental entre escenarios antropizados y silvestres modulan la abundancia de vectores del VNO y el riesgo de transmisión del virus. En una región mediterránea continental en la que la FNO ha emergido recientemente, seleccionamos nueve lugares de muestreo y asignamos a cada uno de ellos tres zonas con un gradiente decreciente de interacción entre fauna silvestre y ganado: A_1_—una granja de caballos donde la interacción es máxima; A_2_—una zona de interacción intermedia a 500–1000 m de la granja; y A_3_—una zona completamente silvestre de baja interacción a 1–5 km de la granja. Con una frecuencia quincenal, estimamos la abundancia de mosquitos en cada uno de los 27 sitios de estudio en mayo‐diciembre de 2018 y abril‐julio de 2019. Estimamos la abundancia de aves y mamíferos, recopilamos información meteorológica y caracterizamos el hábitat de los mosquitos en cada zona. Posteriormente, estudiamos los determinantes de la abundancia de *Culex* spp., *Culex. pipiens* sensu lato (s.l.) Linnaeus, 1758 (Diptera: Culicidae) y *Culex theileri* Theobald, 1903 mediante la construcción de modelos lineales mixtos generalizados binomiales negativos. Identificamos 20 especies de mosquitos, con un notable predominio de *Culex* spp. y, en particular, de *Cx. pipiens* s.l. Encontramos diferencias en la distribución espaciotemporal de la abundancia de *Culex* spp. y confirmamos nuestra hipótesis al hallar importantes efectos de las variaciones ambientales locales en la abundancia. La lluvia acumulada en las quincenas 4 a 14 y la temperatura media de las dos quincenas previas al muestreo se relacionaron de forma positiva y estadísticamente significativa con la abundancia de *Cx. pipiens* s.l. (*Z* = 13.09, *p* < 0.001, y *Z* = 9.91, *p* < 0.001, respectivamente) y de *Culex* spp. (*Z* = 13.35, *p* < 0.001, y *Z* = 6.99, *p* < 0.001, respectivamente), mientras que la temperatura de las dos quincenas previas al muestreo fue un predictor estadísticamente significativo y positivo (*Z* = 14.69, *p* < 0.001) de la abundancia de *Cx. theileri*. El entorno de las granjas fue el más propicio para albergar *Culex* spp. en comparación con los entornos silvestres. Nuestros resultados indican que los entornos mediterráneos continentales son favorables para la circulación y el mantenimiento del VNO, especialmente entornos rurales antropizados como las granjas. Estos resultados repercutirán en la predicción espaciotemporal del riesgo de aparición del VNO en entornos mediterráneos continentales.

## INTRODUCTION

Mosquitoes replicate, maintain and transmit a wide diversity of infectious agents that cause millions of animal and human casualties every year, for example, the microparasites causing human (Battle & Bair, 2021) and avian (Niebuhr et al., 2016) malaria. Approximately 50% of mosquito‐borne human diseases are zoonoses (WHO, 2014), for example, viral meningoencephalitis caused by flaviviruses of the Japanese encephalitis virus (JEV) group, including JEV, West Nile virus (WNV) or Usutu virus (USUV). The relatively recent global spread of WNV, and its enzootic establishment in environmentally favourable settings, has resulted in itself in a worldwide increase in the reporting of West Nile fever (WNF) cases in animals (wild and domestic) and humans with consequent severe health and economic impact (Chancey et al., 2015; Rodríguez‐Alarcón et al., 2021).

Birds play a key role in the maintenance and transmission of WNV, acting both as reservoirs and amplifiers of viral replication (Pérez‐Ramírez et al., 2014). Thus, WNV is maintained in an enzootic cycle that must involve birds that replicate the virus and competent mosquitoes to transmit it to naïve birds (Jeffrey, 2013). Although unlike birds, mammals cannot replicate WNV and infect mosquitoes (Bunning et al., 2002), these could be relevant amplifying hosts along with birds for mammalophilic and plastic mosquito species that use both birds and mammals as feeding resources. Nonetheless, unlike other vectors such as ticks (Peralbo‐Moreno et al., 2022; Ruiz‐Fons et al., 2013), the role of mammals in the diversity and abundance of competent WNV vectors has not been sufficiently explored despite they are abundant in scenarios where mosquitoes, birds, mammals and WNV interact, for example, farm environments, resulting in the emergence of WNF (García‐Bocanegra et al., 2022). It is evident that for a mammal to be infected by WNV, it must be bitten by a female mosquito competent in replicating and transmitting the virus that was previously infected by feeding on the blood of an infected bird, because mammals are not capable of replicating the virus and infecting mosquitoes.

The opportunistic feeding on birds and mammals of the different species of *Culex* spp. mosquitoes (Gómez‐Díaz & Figuerola, 2010), which include the most competent vectors for WNV, makes them a bridge for virus transmission between the avian reservoirs and susceptible mammal species such as horses and humans. The *Culex pipiens* complex is most notable for its wide distribution worldwide (Farajollahi et al., 2011). In Europe, this complex includes three species: (1) *Cx. quinquefasciatus*, which has never been reported in Spain; (2) *Cx. torrentium*, mainly present in cooler environments above 1200 m altitude in Spain (Bueno‐Marí et al., 2012; Delacour‐Estrella et al., 2011); and (3) *Cx. pipiens* sensu lato (s.l.), the most frequent among mosquito populations in Spain (Gangoso et al., 2020). The latter species, in turn, comprises two biotypes—*Cx. pipiens* biotype *pipiens* and *Cx. pipiens* biotype *molestus* (Becker et al., 2010)—which are morphologically indistinguishable, but show behavioural variations (Becker et al., 2012; Fonseca et al., 2004). *Culex pipiens* s.l., together with *Cx. theileri* and some other species of the genus *Culex* that may be locally abundant, for example, *Cx. perexiguus* Theobald, 1903, are the main competent vectors for WNV and USUV transmission in Spain (Bueno‐Marí et al., 2012; Gangoso et al., 2020). Both the structure and composition of mosquito communities vary greatly depending on biotic and abiotic environmental conditions (Ferraguti et al., 2021) that modulate their biology and ecology (Becker et al., 2010; Schlein & Muller, 2012) and, consequently, their abundance (Roiz et al., 2014). These variations in mosquito communities lead, in turn, to spatiotemporal diversity in the risk of transmission of pathogens such as WNV, even at small spatial scales (Paz & Semenza, 2013; Semenza et al., 2016). Describing these variations and understanding their determinants are essential elements for the correct and accurate prediction of the risk of emergence of WNF cases where they emerge with most impact, that is, in equine farms and humanised environments.

Although WNF was the cause of death of a golden eagle (*Aquila chrysaetos*) in south‐central Spain already in 2007 (Jiménez‐Clavero et al., 2008), the disease was not detected in horses and humans until 2010 in very southern Spain. Since then, there has been a geographic and casuistic increase of WNF in animals and humans in two foci, south‐western and north‐eastern Spain (CCAES, 2021). The increasing reporting of WNF cases and the evidence of WNV circulation in southern continental Spain together with previous studies that indicate the widespread presence of *Cx. pipiens* s.l. and *Cx. theileri* in the region, sometimes locally abundant (Durán‐Martínez, 2012, PhD thesis), indicate the need to understand what makes these areas favourable for the presence and establishment of WNV. Thus, the aim of this study was to update information on the composition and distribution of WNV‐competent (and other) mosquito species in central‐southern peninsular Spain within a gradient of wildlife–livestock interactions. We further sought to identify the main abiotic and biotic environmental determinants that modulate the abundance patterns of WNV vectors in wildlife–livestock interaction scenarios.

## MATERIALS AND METHODS

### Study design

**FIGURE 1:**
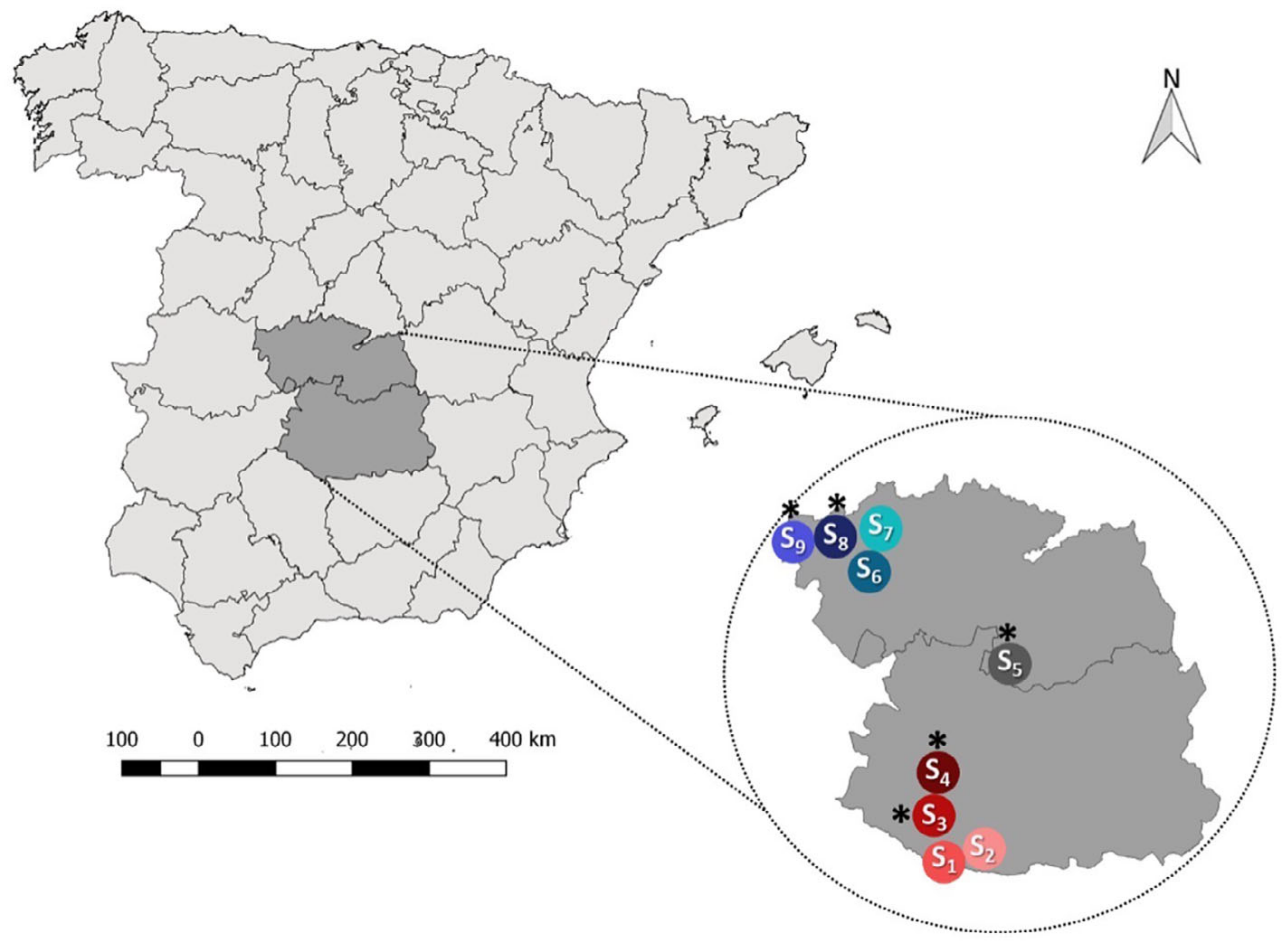
Spatial location of the nine study sites in inland continental Mediterranean Iberia. The asterisks indicate the sites in which bird diversity and abundance estimations were performed.

**FIGURE 2:**
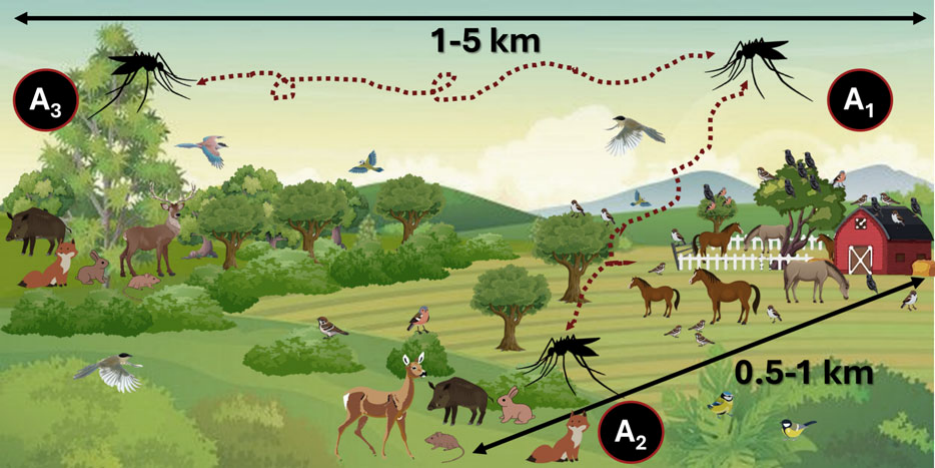
Schematic representation of the scenarios studied in a wildlife–livestock interaction gradient (A_1_—high interaction, A_2_—intermediate interaction and A_3_—low interaction).

We selected nine localities (S_1_–S_9_) in areas of recent WNF emergence and of predominant continental Mediterranean climate (Figure 1) according to the expansion towards central Spain of reported WNF cases (CCAES, 2021), the isolation of the virus in a golden eagle in this region (Jiménez‐Clavero et al., 2008), and the detection of antibodies against WNV in local wild ungulates (Boadella et al., 2012; Casades‐Martí, Cuadrado‐Matías, et al., 2023). To ensure the independence of observations between localities and sampling areas, we considered the mean flight distance of *Culex pipiens* s.l., the most abundant species in the study region (Durán‐Martínez et al., 2012, PhD thesis), which is on average less than 500 m according to capture–mark–recapture studies (Verdonschot & Besse‐Lototskaya, 2014). Four of these localities were in southern Ciudad Real province, in Castilla‐La Mancha (CLM) region, and included the locality where a WNF outbreak in horses was reported in 2014 (CCAES, 2021). In this part of the study region, the minimum straight‐line distance between localities was 12.3 km. Four other localities were selected in the north‐west of Toledo province, also in CLM, close to the locality where an outbreak of WNF was reported in pheasants (*Phasianus colchicus*) in 2015 (CAES, 2021). The minimum distance between localities was 6.3 km for this part of the study region. The last locality was selected in south‐central Toledo province, in the vicinity of the area where the local WNF case was reported in a golden eagle and an additional case was diagnosed in a griffon vulture (*Gyps fulvus*; M. A. Jiménez Clavero, personal communication). The nearest study locality was 56.2 km away from this locality. In each of these localities, we selected three areas where wildlife and domestic animal interaction was variable: (1) A_1_, a horse farm where interaction with birds and other wildlife is frequent; (2) A_2_, as the area of intermediate interaction between wildlife and domestic animals and at a distance of 500–1000 m from the farm; and (3) A_3_, a wild area without livestock and located at a distance of 1–5 km from the selected farm and that *Cx. pipiens* mosquitoes (Verdonschot & Besse‐Lototskaya, 2014), and birds could eventually travel (Figure 2).

### Mosquito survey

We set a mosquito trapping station to each of the three wildlife–livestock interaction scenarios in each locality. A miniature CDC‐type trap (Centre for Disease Control–John W. Hock Company, Gainesville, USA) connected to a 6‐volt gel battery and placed 1.8 m off the ground, and a BG Sentinel trap (BG‐Sentinel 2, Biogents, Germany) placed on the ground connected to a 12 V gel battery, were set per station (see Supplementary Figure 1). Stations were allocated to a maximum of 30 m linear distance from the main water source in the area (stream, river, pond or cattle trough). Traps were operational one night every fortnight between May and December 2018 and again between April and July 2019. We avoided sampling in the coldest months (December–March) when mosquito activity is minimal (Kilpatrick et al., 2006). We used an isothermal container hung 2.2 m above each trapping station and primed it with 600 g of 16‐mm dry ice pellets as a method of slowly dispensing CO_2_ to attract mosquitoes. To avoid the rapid release of CO_2_ on nights of high temperatures, we placed a cold accumulator previously frozen at −80°C in the container. The traps were checked the morning after each sampling night and the trapping insects were transported in humidity‐ and temperature‐controlled containers to our laboratories. After cold stunning, mosquitoes were separated from other insects and female mosquitoes were identified to the species level based on morphological characteristics (Becker et al., 2010) under a stereo microscope (Leica S9D, Leica microsystems, Germany). After identification, they were stored at −80°C. To identify males to species level, the last third of the abdomen was sectioned and subjected to a clarification process by immersing it in Nesbitt liquid (Entomopraxis, Spain) and a hot plate at 30°C for 30 min, after which it was mounted in Berlese fluid (Krantz & Walter, 2009) and the hypopygium structures were observed under a microscope.

### Environmental predictors

#### Habitat favourability

Female mosquitoes need a source of standing water on or near which to lay their eggs (Becker et al., 2010), so the availability of this resource in the vicinity of each trapping station may be an important determinant of mosquito abundance. Similarly, the availability of plants that provide mosquitoes with sugary nutrients and shelter from adverse environmental conditions or predators (also provided by human buildings) could have an impact on local mosquito abundance (Schlein & Muller, 2012). Thus, to estimate the area of water available to mosquitoes (H_0_) and the percentage covered by trees, shrubs and buildings in the vicinity of each trapping station (H_1_) that could provide food/shelter to mosquitoes, we created a 500‐m radius buffer around the coordinates of each trapping station using Google Earth Pro (version 7.3.4). On recent orthophotos (2018–2020) of these areas, each portion of the buffer surface covered with water and tree/shrub/building was digitised and exported in vector format to R software. We thereafter estimated the proportion of the buffer surface occupied by water or mosquito shelter areas.

#### Host availability

*Culex* spp. mosquitoes consume blood from birds and mammals in different proportions depending on the species (Becker et al., 2010). Diet studies of *Cx. theileri* females classify them as mammalophilic (Demirci et al., 2014), while most diet studies in *Cx. pipiens* s.l. indicate ornithophilic food preferences (Hamer et al., 2009). However, of the two biotypes defined for this last species, *Cx. pipiens pipiens* Linnaeus, 1758 is considered strictly ornithophilic, while *Cx. pipiens molestus* Forskål, 1775 is considered anthropophilic (Becker et al., 2012; Fonseca et al., 2004). Hybridization between the two biotypes is frequently found in Spain (e.g., Bravo‐Barriga et al., 2017; Fonseca et al., 2004; Frías et al., 2022; Rudolf et al., 2013), resulting in a wide range of hosts on which *Cx. pipiens* s.l. can feed. Thus, both birds and mammals can serve as food to WNV vectors.

To estimate the availability of mammalian hosts for mosquitoes, we attached a camera trap (TROPHY CAM HD, Bushnell Outdoor Product, Kansas, USA) near each trapping station to a wooden stick or tree trunk at a height of 40 cm from the ground (Hofmeester et al., 2017). Indicators (wood sticks or stone piles) were placed 5 and 10 m linearly from the focal centre of the camera to monitor variations in the detectability of mammals as a function of their size (Palencia et al., 2021). The correct daily functioning of the cameras was monitored by programming the automatic capture of two images at specific times of the day and night. The cameras were active for three fortnights during the study period, including one in summer and one in autumn 2018, and a third in spring 2019. Images recorded in each fortnight were downloaded and visualised to separate them by species (Supplementary Figure 2). If two species were observed in the same image, the image was duplicated to be considered as an image for both. In the case of ungulates, we only considered those images taken within 10 m of the camera, which was reduced to 5 m for smaller animals. To estimate the time of use of the space in front of the camera and employ this as an indicator of mammal availability (relative abundance) for mosquitoes, we followed the protocol previously described by Cuadrado‐Matías et al. (2024); a similar, although more qualitative, approach was employed by Fikrig et al. (2022) for *Aedes albopictus* Skuse, 1894 (Diptera: Culicidae). We separated the images of each species taken by the camera into visits. When two contiguous images of the same species were recorded with an interval of more than 10 min, they were considered to belong to different visits. After this classification, we estimated the duration of each visit (in minutes) and the maximum number of animals captured during the visit; in case of more than one animal, the number was multiplied by the duration of the visit. Finally, the times for each species were summed over the total of the three sampling fortnights for each chamber, and an index of this time with respect to the total time (in minutes) that each camera was active was employed as an indicator of availability/abundance.

To estimate bird species abundance, biannual censuses were carried out in 15 of the study areas during the mosquito season (spring and autumn). A 1‐km‐long transect per area was designed to run close to the main water source where the trapping station was set. The transect was divided into 10 consecutive stretches of 100 m in length. Four bird sighting and listening stations were distributed along each 1 km long transect with a separation of 300 m. Two dawn and two dusk counts per census were carried out. Two experienced researchers in bird visual and audio identification walked at a slow path along each transect carrying binoculars, recording the birds sighted or heard on each 100 m stretch both within a 25 m distance band perpendicular to the transect path (25 m in each side of the transect) and outside that band (Bibby et al., 2000). The same experienced observers conducted bird counts for 10 min at each station, recording birds within and outside a 25 m radius from the observer. To minimise double counts, we established a temporal separation between stations and transect stretches, and also between consecutive stretches, of 5 min. Bird abundance was estimated as the total sum of birds sighted or heard within 25 m from the observer at each of the four stations. The final abundance per area was estimated as an average of the values for each count conducted.

#### Weather determinants

**FIGURE 3:**
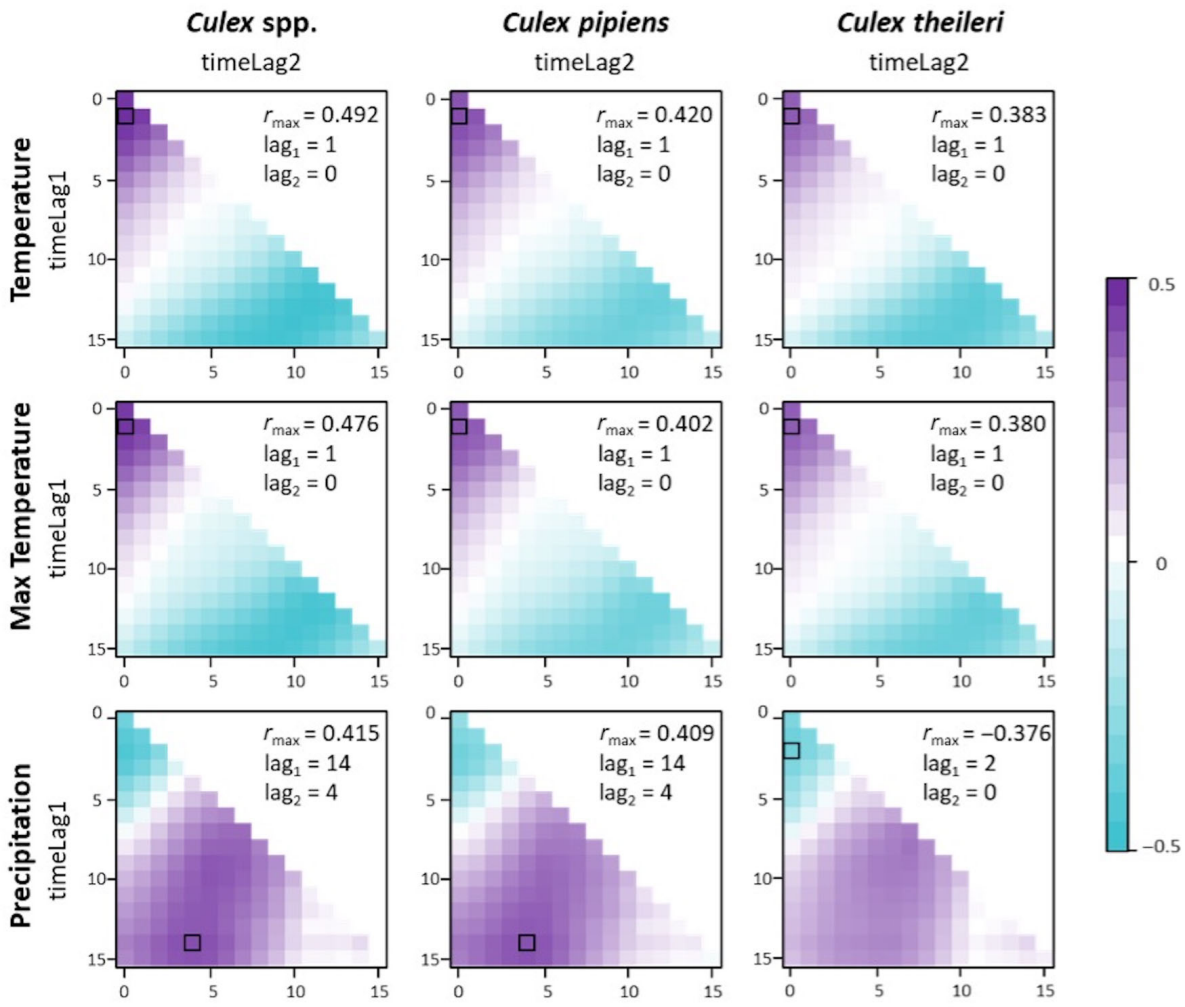
Cross‐correlation matrices (CCMs) displaying the time lagged Spearman correlation coefficient between West Nile virus vector abundance (*Culex* spp., *Cx. pipiens* sensu lato and *Cx. theileri*) and weather quantities (mean and maximum temperature [°C] and accumulated rainfall [mm]) at a fortnight temporal scale. Black squares in each chart show the highest correlation coefficient observed and employed to select the time lag at which the predictors should be considered for statistical modelling.

Abiotic environmental conditions modulate mosquito population dynamics (Brugueras et al., 2020). Therefore, we estimated weather variability in each study locality from daily data recorded from 2017 to 2019 by local weather stations selected from the Spanish Meteorological Agency (AEMET) weather station network. The meteorological variables that we calculated as potential predictors of mosquito population dynamics included (1) the average value of the fortnightly mean daily temperature, (2) the average daily maximum temperatures in the fortnight and (3) the accumulated precipitation in the fortnight. The association of time‐lagged environmental weather quantities to the fortnight dynamics of *Culex* spp., *Cx. pipiens* s.l. and *Cx. theileri* abundance was explored by cross‐correlation matrices, a useful method to visualise lagged weather effects on mosquito abundance (Curriero et al., 2005). Cross‐correlation matrices gather the bivariate relationships of a time series response variable (e.g., mosquito abundance) with time series explanatory variables (e.g., weather quantities) by allowing the lag effect of the explanatory variable to extend over different time periods of variable length with respect a given time of the response variable (see Groen et al., 2017). Spearman's rank order correlations between mosquito abundance and weather conditions for a given period and time lag were displayed as cross‐correlation maps (Figure 3). Time lags were expressed at a fortnight temporal scale, and the maximum lag considered was 15 fortnights (7.5 months). The selection of this maximum lag responds to the expected dependence of mosquito population dynamics at a particular time of year on past conditions (Torina et al., 2023; e.g., a severe winter may increase mortality of overwintering adults and decrease the number of individuals with which an annual reproductive cycle begins) or those that occur over long periods (e.g., rainwater accumulation occurs over several months of the year and conditions the availability of breeding sites for mosquitoes several months later).

### Data analyses

The data analysis was conducted in two phases. In the initial phase, we explored the nature of the estimated predictors (Table 1), the relationships between them and with the response variable, and the presence of potential imbalances in the cases in accordance with recommendations for descriptive exploratory analyses (Zuur et al., 2010). From this initial process, we discarded some predictors that were highly correlated with other predictors (correlation coefficient ≥|0.6|) and presented a poorer statistical relationship with the response variables (Table 1). We thereafter searched for the determinants of WNV vectors both (1) holistically, that is, considering all captured species of the genus *Culex* as a whole (due to the potential plastic behaviour of the two most abundant *Culex* spp. in our study region and because the goal of our study was on WNV competent vectors as a whole), and (2) specifically, for each of the two most abundant species of the genus, *Cx. pipiens* s.l. and *Cx. theileri,* because they could be not extremely plastic in host selection and their ecology/behaviour be widely diverse, thus playing differential roles in WNV transmission in our study locations. We employed time‐lagged weather conditions (selected on the basis of the maximum correlation coefficient observed between the parameter and the response variable in CCMs) as predictors of the fortnight variation in mosquito abundance (Figure 3). Due to logistic constraints, we were unable to estimate bird abundance at four of the nine study locations, so the exploration of the role of bird abundance in mosquito population dynamics was done only using data from the five sampled locations.

**TABLE 1:** Predictors of West Nile virus vector abundance estimated for statistical modelling and range values.

Predictor group	Predictor acronym	Description	Variable type (range)
Spatial	**S**	Survey location	Categorical (1–9)
	**igrad**	Interaction gradient	Categorical (1–3)
Habitat	**H**_**0**_	Proportion of buffer surface covered with water	Continuous (0%–17.5%)
	**H**_**1**_	Proportion of buffer surface covered with buildings, trees and shrub	Continuous (11.8%–82.8%)
Abiotic	**AT.0_1**	Average temperature of sampling and preceding fortnights	Continuous (8.6–28.8°C)
	AMT.0_1	Average maximum temperature of sampling and preceding fortnights	Continuous (13–37.5°C)
	**AR.4_14**	Accumulated rainfall along fortnights 4 to 14 prior to sampling	Continuous (0–445.1 mm)
	**AR.0_2**	Accumulated rainfall along the three last fortnights to sampling	Continuous (30.4–811.4 mm)
Biotic	**ung.ab**	Ungulate abundance index	Continuous (0.00–0.06)
	bird.ab	Total bird abundance index	Continuous (4.2–170)

*Note*: Predictors selected for modelling after a descriptive exploratory analysis are shown in bold type letter case.

The models were built under the hierarchical control of sampling location, which was considered as a random factor. The variation in the scales of measurement of the different predictors was reduced by logarithmic transformation of the predictors prior to the construction of the statistical models. All possible negative binomial regression models for the predictor combinations considered (Table 2; Supplementary Table 1) were constructed using the free R software package ‘lme4’. Models were ranked according to the corrected Akaike information criterion (AIC) and those with an AIC difference (ΔAIC) of less than two were combined to obtain an average model using the ‘dredge’ and ‘model.avg’ functions of the R package ‘MuMin’ (Barton, 2009). The resulting average model was considered as the model with the best fit to explain variation in WNV vector abundance. We estimated the proportion of the deviance in the response variables that each of the selected models explained with respect to the null model as an indicator of their relevance in explaining the population dynamics of *Culex* species in our conditions.

**TABLE 2:** Output of average selected model including the selected predictors (abbreviations as shown in Table 1), the estimate and its associated standard error (SE), the statistic (*Z*), the *p*‐value and the explained deviance (ED).

Model set	Predictor	Estimate	SE	*Z*	*p*	ED (%)
*Culex* spp.	Intercept	−17.7028	11.034	16.009	***	57.01
	ung.ab	9.6391	88.945	1.083	0.279	
	H_1_	−3.0878	0.6622	4.654	***	
	igrad				*	
	A_1_	Ref.	‐	‐		
	A_2_	0.4665	0.1756	2.651	**	
	A_3_	0.2908	0.2181	1.330	0.183	
	AR.4_14	1.8965	0.1445	13.094	***	
	AT.0_1	2.7720	0.2791	9.910	***	
*Culex pipiens*	Intercept	−17.9564	1.2209	−14.708	***	59.63
	H_1_	−2.7307	0.5880	−4.644	***	
	igrad				**	
	A_1_	Ref.	‐	‐		
	A_2_	0.5868	0.1748	3.358	***	
	A_3_	0.3442	0.2276	1.513	0.130	
	AR.4_14	2.1507	0.1611	13.353	***	
	AT.0_1	2.1376	0.3055	6.996	***	
*Culex theileri*	Intercept	−16.17205	1.25600	12.864	***	66.33
	H_1_	−2.17226	0.39402	5.501	***	
	AR.0_2	0.05440	0.05498	0.989	0.323	
	AT.0_1	5.33777	0.36280	14.699	***	
	igrad				0.239	
	A_1_	Ref.	‐	‐		
	A_2_	−0.08782	0.15290	0.574	0.566	
	A_3_	0.01507	0.11053	0.136	0.892	

* *p* < 0.05;

** *p* < 0.01;

*** *p* < 0.001.

## RESULTS

### Descriptive findings

A total of 4343 mosquitoes of 20 different species were captured during the sampling period. Of these, 3864 were females, 390 were males and 89 could not be sexed. Of the total number of mosquitoes, 3796 belonged to the genus *Culex* (87.4%), 151 to the genus *Culiseta* (*Cs*.) (3.5%), 150 were *Aedes* spp. (3.5%), 59 were *Anopheles* spp. and 1 was identified as *Orthopodomyia (Or.) pulcripalpis* Rondani, 1872 (Diptera: Culicidae) (Supplementary Table 2). The genus could not be determined morphologically in 186 mosquitoes due to the poor condition of the exoskeletons (Table 3). Of the 3796 *Culex* spp. specimens, 3151 were identified as *Cx. pipiens* s.l., including 2950 females and 201 males. The species *Cx. theileri* accounted for 630 of the *Culex* spp. captured, including 587 females and 43 males. In addition, seven *Cx. perexiguus*, five *Cx.hortensis*, Ficalbi, 1889, two *Cx. laticinctus* Edwards, 1913 and one *Cx. modestus* Ficalbi, 1890, were captured (Supplementary Table 2).

**TABLE 3:** Total number of captured mosquitoes throughout genus, study site and interaction gradient, with specific detail of the numbers of *Cx. pipiens* sensu lato and *Cx. theileri*.

Site	Area	*Culex pipiens*	*Culex theileri*	*Culex* spp.	*Cs*. spp.	*An*. spp.	*Ae*. spp.	Undt.	Others	Total
S_1_	A_1_	4	0	4	2	0	1	0	0	7
	A_2_	8	1	11	0	2	0	0	0	13
	A_3_	1	1	2	1	0	0	1	0	4
Subtotal S_1_		13	2	17	3	2	1	1	0	24
S_2_	A_1_	2	12	15	7	0	3	5	0	30
	A_2_	2	20	22	3	1	3	2	0	31
	A_3_	2	5	7	1	0	0	3	0	11
Subtotal S_2_		6	37	44	11	1	6	10	0	72
S_3_	A_1_	33	17	51	0	0	1	1	0	53
	A_2_	117	63	180	1	3	4	12	0	200
	A_3_	2	3	5	0	0	0	2	0	7
Subtotal S_3_		152	83	236	1	3	5	15	0	260
S_4_	A_1_	19	61	80	0	14	0	3	0	97
	A_2_	23	29	52	1	7	2	2	0	64
	A_3_	36	41	77	3	3	3	10	0	96
Subtotal S_4_		78	131	209	4	24	5	15	0	257
S_5_	A_1_	77	35	112	3	1	12	3	0	131
	A_2_	34	5	39	1	0	1	1	0	42
	A_3_	22	3	26	0	1	6	5	0	38
Subtotal S_5_		133	43	177	4	2	19	9	0	211
S_6_	A_1_	419	47	468	49	0	2	12	0	531
	A_2_	1210	1	1213	9	2	4	32	0	1260
	A_3_	235	40	276	12	5	12	7	0	312
Subtotal S_6_		1864	88	1957	70	7	18	51	0	2103
S_7_	A_1_	248	6	255	28	2	0	30	0	315
	A_2_	89	8	97	5	1	2	6	0	111
	A_3_	72	4	77	4	0	3	5	0	89
Subtotal S_7_		409	18	429	37	3	5	41	0	515
S_8_	A_1_	1	17	18	1	0	8	3	0	30
	A_2_	14	0	14	0	0	2	2	0	18
	A_3_	28	15	43	2	3	21	2	0	71
Subtotal S_8_		43	32	75	3	3	31	7	0	119
S_9_	A_1_	70	66	138	7	3	16	7	0	171
	A_2_	171	30	202	3	6	21	11	0	243
	A_3_	212	100	312	8	5	23	19	1	368
Subtotal S_9_		453	196	652	18	14	60	37	1	782
All	A_1_	873	261	1141	97	20	43	64	0	1365
	A_2_	1668	157	1830	23	22	39	68	0	1982
	A_3_	610	212	825	31	17	68	54	1	996
Total		3151	630	3796	151	59	150	186	1	4343

Abbreviations: *Ae*., *Aedes* spp.; *An*., *Anopheles* spp.; *Cs*., *Culiseta* spp.; Undt., undetermined genus.

### Spatiotemporal abundance patterns

**FIGURE 4:**
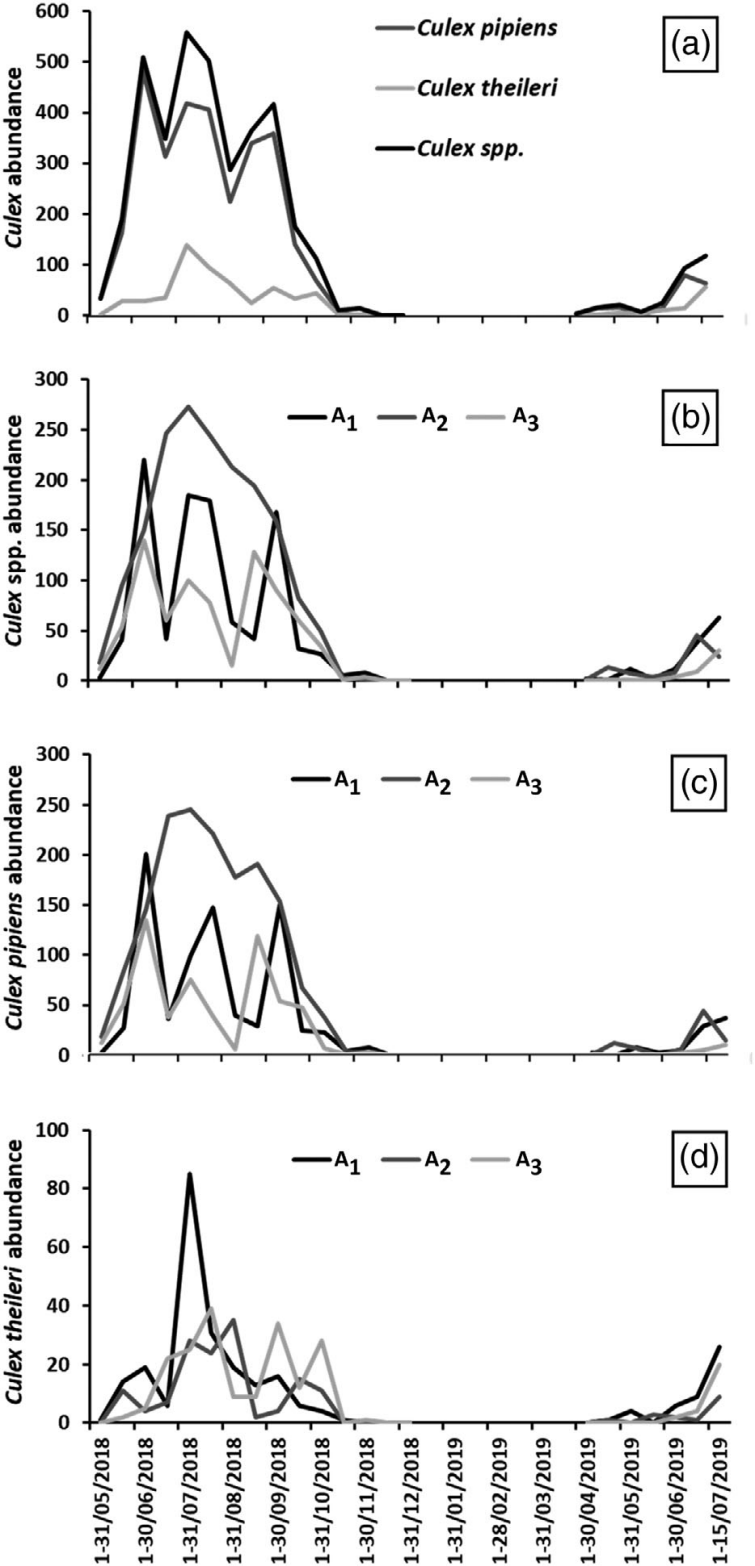
Graphical representation of the evolution of West Nile virus vector captures at a fortnight temporal scale from May 2018 to July 2019 (no surveys were carried out in January to March 2019). Chart ‘A’ displays the summed number of captures (nine sites) for *Culex* spp., *Cx. pipiens* sensu lato (s.l.) and *Cx. theileri*, whereas charts ‘B–D’ display the summed number (nine sites) of *Culex* spp., *Cx. pipiens* s.l. and *Cx. theileri*, respectively, throughout the scenarios of variable wildlife–livestock interaction (A_1_–A_3_).

We observed great diversity in both the number of captures of mosquitoes and the composition of mosquito communities in the sampling localities and in the wildlife–livestock interaction scenarios (Table 2). The highest abundances were observed at localities in the north‐west of the study area, in S_6_ and S_9_, and in general, although with slight local variations, in the intermediate wildlife–livestock interaction area (A_2_). Species of the genus *Culex* were predominant in the nine study localities, representing between 61.1% and 93.1% of the captures (Supplementary Table 2). The predominance of *Cx. pipiens* s.l. over *Cx. theileri* was evident in six of the nine localities, except for S_2_, S_4_ and S_8_ where the predominance pattern was reversed. *Culex perexiguus*, an ornithophilic mosquito highly competent in WNV replication and transmission, was captured in low abundance in S_3_, S_6_, S_7_ and S_9_. We observed higher species diversity in S_6_–S_9_ (9–11 species) compared with S_1_–S_5_ (6–8 species) (Supplementary Table 2). The highest diversity in S_6_–S_9_ was observed in species of the genera *Culiseta* and *Aedes*. The highest number of total captures of *Culex* spp. (*n* = 1957 specimens) was observed in S_6_. It was followed in order of abundance by S_9_ (*n* = 652), S_7_ (*n* = 429), S_3_ (*n* = 236), S_4_ (*n* = 209), S_5_ (*n* = 177), S_8_ (*n* = 75), S_2_ (*n* = 37) and S_1_ (n = 17) (see Table 2). As in the overall count, the areas of the highest abundance of *Culex* spp. (consequently *Cx. pipiens* s.l.) were those around the farms, A_2_ and A_1_, respectively, with a lower overall abundance in the wildest area (A_3_), with exceptions such as S_4_, S_8_, and S_9_. However, *Cx. theileri* prevailed in A_1_, followed by A_3_ and A_2_, respectively (Supplementary Table 3; Figure 4).

As shown in Figure 4 and Supplementary Figure 3, the highest number of *Culex* spp. captures in 2018 was obtained between the second half of May and the first half of October, at which time mosquito abundance declined drastically until December. In 2019, abundance values began to rise markedly later, between the second half of June and the first half of July, obtaining lower values than in 2018 for the same period. We observed abundance peaks of *Cx. pipiens* s.l. between June and September, while *Cx. theileri* presented an overall abundance peak in July within a sustained abundance within its activity period (May–October). Despite the higher abundance of *Cx. pipiens* s.l., both *Cx. pipiens* s.l. and *Cx. theileri* showed similar activity patterns in all sampling localities except S_2_ and S_4_ (Supplementary Table 3).

### Drivers of WNV vector abundance

The temperature‐related predictor that showed the highest statistical relationship with the abundance of *Culex* spp., *Cx. pipiens* s.l. and *Cx. theileri* in CCMs was the average of the daily mean temperature values of the sampling and previous fortnights (Table 2; Figure 3). For *Culex* spp. and *Cx. pipiens* s.l., the rainfall‐related predictor with the highest statistical association with their abundance was the accumulated rainfall between the 14th and 4th fortnights prior to sampling, while for *Cx. theileri* it was the rainfall accumulated along the sampling fortnight and the two previous ones (Figure 3). The maximum correlation coefficients for temperatures (both mean and maximum) were low and positive in value, while those for cumulative rainfall were positive for *Culex* spp. and *Cx. pipiens* s.l. and negative for *Cx. theileri* (Figure 3).

The best model for *Culex* spp. was able to explain 57.01% of the total deviance in fortnight abundance, while this proportion increased to 59.63% in the *Cx. pipiens* s.l. model and 66.33% in the *Cx. theileri* abundance model. Of the models constructed for *Culex* spp. mosquitoes according to our focus on WNV global transmission risk, two best models explained the fortnightly variation in abundance (ΔAICc < 2; Supplementary Table 1). The average model output resulting from these two models is indicated as relevant predictors: (1) the local abundance of ungulates (positive and not statistically significant), (2) the proportion of ground covered by habitat potentially favourable for mosquito (plant)feeding and shelter (negative and statistically significant), (3) the sampling area (positive and statistically higher in A_2_ than in reference category A_1_), (4) cumulative rainfall (positive and statistically significant) and (5) temperature (positive and statistically significant) (Table 3). We did not observe an effect of local bird abundance on mosquito abundance in the best‐fit model (Table 3).

The average model to explain the fortnight variation in *Cx. pipiens* s.l. abundance showed a very similar output to the model for *Culex* spp., with the exception that local ungulate abundance was not a predictor retained by the best‐fit models. For *Cx. theileri*, the average model included predictors related to habitat, sampling area, rainfall and temperature, although the differences observed between sampling areas and in relation to the rainfall predictor were not statistically significant. No bird abundance effects were either observed on *Cx. pipiens* s.l. or *Cx. theileri* (Table 3).

## DISCUSSION

### Mosquito community structure in continental Mediterranean Spain and implications

Our study confirms the predominance of *Culex* species in western continental Mediterranean Spain, in agreement with previous results for CLM region (Durán‐Martínez, 2012, PhD thesis) and the higher abundance of *Cx. pipiens* s.l. compared with *Cx. theileri* and other *Culex* species. Undoubtedly, this result indicates the environmental favourability for the potential enzootic establishment of zoonotic (WNV, USUV) and non‐zoonotic (Bagaza virus, BAGV) flaviviruses for which these mosquitoes are vectors, and the potential emergence of human/porcine JEV encephalitis cases in case of JEV introduction. Given the potential and demonstrated opportunistic feeding habits of some *Culex* spp., including birds and mammals (Becker et al., 2010; Demirci et al., 2014), and the joint presence of *Cx. pipiens* s.l. and *Cx. theileri* in all study localities, we can conclude that WNV may be transmitted between reservoirs (birds) and susceptible mammals (horses and humans) by bridge vectors with plastic feeding habits. Indeed, as only birds replicate WNV, there must necessarily be mosquito vector species/individuals with sufficient plasticity in host selection to become infected after feeding on a bird to transmit the virus to a mammal. If we add to this fact the expected presence of both *Cx. pipiens pipiens* and *Cx. pipiens molestus* (and their hybrids) biotypes (Bravo‐Barriga et al., 2017; Fonseca et al., 2004; Frías et al., 2022; Rudolf et al., 2013) together with their abundance in wild, domestic and mixed environments, the existence of bridge vectors could be the reason behind the occurrence of WNF outbreaks in the area (CCAES, 2021; Jiménez‐Clavero et al., 2008). In 2023, two cases of WNF were reported in CLM (ECDC, 2024), the first human case in the region and an outbreak in captive birds, 47 and 16 km, respectively, away from one of our sampling points in Toledo (S_6_).

After the genus *Culex*, species of the genus *Culiseta* were the second most abundant group of mosquitoes in the study areas. Although the vectorial capacity of Iberian *Culiseta* spp. for flaviviruses is uncertain (Martinet et al., 2019), *Cs. incidens* Theobald, 1901 (Diptera: Culicidae) (Reisen et al., 2006) and *Cs. inornata* Williston, 1893 (Goddard et al., 2002) were confirmed as competent WNV vectors, so the role of Iberian *Culiseta* spp. in WNV ecology should be better understood. In contrast to previous data in CLM (Durán‐Martínez, 2012, PhD thesis), the third most abundant group of mosquitoes in our study were those of the genus *Aedes* (*Ochlerotatus*). Unfortunately, many of the captured specimens of the genus *Aedes* could not be identified to the species level due to conservation and handling damage and shall be subject to molecular classification in the future. As shown in Supplementary Table 2, several of the *Aedes* species, together with *Or. pulcripalpis* and some species of the genus *Culex* found in the study region had not been previously documented (Bueno‐Marí et al., 2012; Durán‐Martínez, 2012, PhD thesis). Possibly, these first records, particularly some *Culex* and *Aedes*/*Ochlerotatus* species such as *Cx. perexiguus*, *Cx. modestus*, *Ae. caspius* Pallas, 1771, *Ae. berlandi* Séguy, 1921/*pulcritarsis* Rondani, 1872 or *Ae. geniculatus* Olivier, 1791, are a consequence of the scarce studies that have been carried out in this region of Spain or perhaps the consequence of real changes in mosquito communities (see Casades‐Martí et al., 2022). An interesting finding was the presence of *Cx. perexiguus* in localities where WNV outbreaks have been reported in horses (S_3_) or in those close to the areas with reported WNV circulation in domestic birds and horses (S_6_, S_8_ and S_9_) (CCAES, 2021). However, the low abundance of the species suggests that its role in these continental environments should be limited in contrast to thermo‐Mediterranean‐dominated landscapes in southern Spain (Cuervo et al., 2022; Vázquez et al., 2011) or perhaps limited to large extensions of inland aquatic ecosystems.

### Environmental factors associated with WNV vector abundance

#### Methodological considerations for the interpretation of findings

Our initial design comprised biweekly sampling of mosquitoes between April and December 2018 and 2019, but for logistical reasons associated with the funding, we were unable to begin sampling until May 2018 and could not extend it to December 2019. Mosquito population dynamics is highly dependent on prevailing environmental conditions, and these vary between years (see Supplementary Fig. 1 in Casades‐Martí, Holgado Martín, et al., 2023). However, our approach to analysing the relationship between abiotic constraints and biweekly abundance was temporally explicit across the time window in which abiotic constraints can condition mosquito population dynamics, and thus we did not consider it relevant to analyse only abundance dynamics in 2018, in which we had sampled almost a full cycle of activity. The selection of the lagged time window of 15 fortnights was based on the expected biology of *Culex* spp. in climatic temperate regions (Becker et al., 2010). The annual cycle starts at the end of winter with adult females that survived the adverse winter conditions of continental Spain. Overwintering females become active with the rising spring temperatures and start searching for hosts. After a first egg laying in spring, when temperatures are warm and the accumulated winter–spring rainfall offers the availability of breeding sites, population growth begins to become exponential. Under appropriate environmental conditions, including the persistence of suitable spots for the development of new generations, the population continues its exponential growth. As temperatures increase, intergenerational time decreases, and abundance increases until reaching a maximum peak in the warmer months of the year, when the high temperatures impair adult survival (Ciota et al., 2014). The intense summer drought of Mediterranean Spain results in a decreasing availability of breeding sites towards autumn, which, together the decreasing autumn temperatures, cause the activity of adult specimens to decrease as they seek for winter refuge. Thus, the summer abundance peaks might be highly dependent on the number of females that survived the winter and the water accumulated after the main period of rains in winter–spring, about 7–8 months before (approx. 15 fortnights).

An essential aspect to consider in interpreting our findings is the method used to estimate an indicator of mammalian availability/abundance for mosquitoes. When the spatial scale of the study is equal to or larger than the home range of the mammals being studied, the use of strategies focused on estimating the density of each species may be the most appropriate (e.g., Acevedo et al., 2007; Palencia et al., 2022, at the scale of tens to hundreds of hectares). However, when this scale decreases, it is advisable to use an indicator of abundance (e.g., Hofmeester et al., 2017 at a scale of 1 ha), especially at scales of few meters (e.g., Cuadrado‐Matías et al., 2024 at a scale of less than 20 m). Although we do not know the spatial range of attraction of carbon dioxide‐supplemented light traps for mosquitoes, one estimate for *Culicoides* spp. indicates an effective radius of about 15.3 m (Kirkeby et al., 2013). Assuming that for mosquitoes this radius could be extended to 50 m due to their larger size and flight speed, we would be covering an area between 0.1 and 0.8 ha. A single camera trap is perhaps insufficient to estimate host availability for mosquitoes even if sampling is extended over time, so perhaps the abundance index underestimates the actual host availability for mosquitoes. Even so, the aim of our study was to compare with different scenarios in which the estimate was made with the same approximation, so we believe that the results obtained are still valid despite the potential underestimation of actual host availability. It is most likely that our approximation has underestimated to a greater extent the less abundant mammal species or those more difficult to detect by cameras, such as carnivores or lagomorphs, so that both a greater number of cameras and a longer recording time would be advisable in similar approximations in the future.

#### Environmental drives of WNV vector abundance

As expected, due to the predominance of *Cx. pipiens* s.l. within the genus *Culex*, model output for the genus and the species was very similar. We believe that for the purpose of our study, knowing the determinants of WNV vector abundance, it is essential to consider all *Culex* species as a whole because we do not know the precise role that each of them plays in the life cycle of the virus in continental Mediterranean Spain. Therefore, even though different *Culex* species present very particular life cycles and biologies that determine different ecologies and require specific analysis (as we did in this work), we consider it important to estimate which environmental factors are associated with a higher overall abundance of *Culex* spp.

Although our hypothesis about the influence of host abundance on WNV vector abundance was that both bird and mammal would be good *Culex* spp. abundance predictors, we only confirmed a non‐statistically significant effect of ungulate abundance for *Culex* spp., but neither for the predominantly ornithophilic *Cx. pipiens* nor for the mammalophilic *Cx. theileri* was that effect confirmed. Although the biotype *Cx. p. molestus* is more abundant in anthropized environments, as demonstrated by Bravo‐Barriga et al. (2017), these authors found no variation in the frequency of hybrids between *Cx. p. pipiens* and *Cx. p. molestus* in urban, rural or wild environments. It would have been expected that, under the assumed predominance of *Cx. p. molestus* in the farms and a similar abundance of hybrids of molestus and pipiens forms *and Cx. theileri*, all of them capable of feeding on mammals or with preferences for mammals, we would have observed some effect of ungulate abundance, especially because this was higher in the farms (Supplementary Table 4). We observed no association (data not shown) between the specific abundance of ungulates on farms (A_1_) and the abundance of *Cx. pipiens* s.l. or *Cx. theileri*. The fact that ungulate abundance was higher in farms (Index = 0.0096) than in intermediate interaction areas (A_2_; Index = 0.0028) and somewhat higher than in wild areas (A_3_; Index = 0.0049), with higher average abundances of *Culex* mosquitoes in intermediate interaction zones, especially of *Cx. pipiens* s.l., may explain this result. A more precise characterisation of the mammal community structure in the future could shed light on the role that these vertebrates may play in *Culex* spp. population dynamics, especially if accompanied by studies of local host preferences. More striking was the absence of an effect of local bird abundance on *Cx. pipiens* s.l. abundance, because it would be expected to be a good local predictor of the abundance of this ornithophilic species. We included an estimate of the total bird abundance and not specific bird order, for example, Passeriformes, abundance estimates because we did not expect a clear pattern of a specific selection of some orders of birds over others by ornithophagous *Culex* spp. (González et al., 2020). While the trap placement height (maximum 1.8 m) could potentially account for the findings, previous studies have not consistently observed a significant effect of height on the capture of ornithophilous species like *Cx. pipiens* (Darbro & Harrington, 2006; Drummond et al., 2006). Furthermore, in the study areas, characterised by low shrubland and predominance of *Quercus* spp. that do not exceed 7 meters in height (Galiano et al., 2012), this factor was not deemed particularly influential. The diversity of passerines modulates the risk of exposure to flaviviruses of birds in the study area (Casades‐Martí, Holgado Martín, et al., 2023), but clearly because susceptibility to flaviviruses is higher for passerines than for other bird orders (Pérez‐Ramírez et al., 2014). Complementing vector abundance with the prevalence of *Flavivirus* infection may offer important advantages in understanding the relationship between vector/*Flavivirus* population dynamics and host community structure in the future. Moreover, passerine and total bird abundance variables showed a positive and high correlation coefficient (*r* > 0.8; data not shown), but the one that showed the strongest association with *Culex* spp., *Cx. pipiens* s.l. and *Cx. theileri* abundance in the initial exploratory analysis was total bird abundance. Maybe the potential plastic‐feeding behaviour of these mosquitoes is behind the lack of a clear host abundance effect once a minimum abundance is achieved.

The inclusion of predictors related to favourable habitats for mosquitoes was based on the hypothesis that the availability of breeding and shelter/feeding areas could influence abundance. Contrary to this, the results showed a negative association between the proportion of ground covered by trees/shrubs/buildings and the abundance of *Culex* spp., *Cx. pipiens* s.l. and *Cx. theileri*. This statistical finding clearly derives from the lower average tree/shrub/building cover observed in farms (0.299) compared with that of A_2_ (0.415) and A_3_ (0.515) areas. Our habitat estimate cannot consider the qualitative contribution that plant community composition might have on mosquitoes, where perhaps many Mediterranean plant species do not provide relevant trophic or refuge resources to mosquitoes. It would be necessary to understand which plant species in Mediterranean ecosystems offer resources usable by mosquitoes to investigate the potential role that habitat structure and composition may have on their population dynamics. The selected localities were non‐urban areas (except S_6_ and S_7_ which were on the outskirts of a big city), which perhaps conditioned both the expected abundance of *Culex* spp.—more abundant in anthropized environments, especially in the case of *Cx. pipiens* (Becker et al., 2010; but see Ferraguti et al., 2016)—and the large variation in the local proportion of presumed favourable habitat (12%–83%). Meanwhile, the local area covered by water did not affect mosquito local abundance, but maybe one‐year sampling does not allow capturing the influence of breeding site availability at this temporal scale. Also, the fact that the sampling was conducted purposely in areas with year‐permanent water sources may hinder any positive effect of the local variation in the water‐covered surface.

The highest abundance of *Culex* spp. and *Cx. pipiens* s.l. in areas of intermediate‐to‐high wildlife–livestock interaction agreed with the higher exposure risk to flaviviruses of wild birds in the farm environment (Casades‐Martí, Holgado Martín, et al., 2023). As discussed by Casades‐Martí, Holgado Martín, et al. (2023), the farm environment (A_1_) and the immediate neighbourhood (A_2_) may be important attractants for passerines, resulting in a greater aggregation of these birds and, consequently, facilitating the transmission of flaviviruses between mosquitoes and birds. However, bird abundance is neither associated with *Culex* spp. abundance (this study) nor with the likelihood of birds having antibodies to *Flavivirus* (Casades‐Martí, Holgado Martín, et al., 2023). Bird community structure and spatial aggregation of some species may perhaps be an important modulator of the risk of virus transmission. However, the differential role that bird species play as hosts for *Culex* spp. mosquitoes is poorly understood, and we assumed that they may similarly contribute to feeding ornithophilic mosquitoes. Interestingly, the two most abundant bird species on the farms in our study (Supplementary Table 5), the house sparrow (*Passer domesticus*) and the spotless starling (*Sturnus unicolor*), presented variable spatial distribution patterns. While the house sparrow, a good reservoir of WNV (Pérez‐Ramírez et al., 2014), was more abundant on farms (Abundance Index (AI) = 8.9) than in its neighbourhood (AI(A_2_) = 2.3) or in wild areas (AI(A_3_) = 1.2), spotless starling abundance was on average higher in A_2_ (AI = 28.8) than on farms (AI(A_1_) = 18.0) and in wild environments (AI(A_3_) = 0.8). Although there is very little information on the role that the spotless starling may play as a reservoir of WNV, its European migratory relative, the common starling (*Sturnus vulgaris*), seems not to be a good reservoir of WNV (Cabe, 2021). However, the behaviour and distribution of the common starling differs widely from that of the spotless starling, a resident species in the western Mediterranean with a high affinity for anthropized environments. Given that house sparrow and spotless starling are the two most abundant birds both in horse (this study) and poultry farms (Sánchez‐Cano et al., 2024), it seems essential to find out what role these two birds have as reservoirs of WNV and hosts of *Culex* spp. The higher abundance of *Culex* spp. in A_2_ could thus be a consequence of a higher aggregation of birds in this setting compared with farm and wild environments (Supplementary Table 5), but given the lack of influence of bird abundance observed in this study, it could rather be a consequence of *Culex* spp. community structure. In intermediate environments between anthropized and wild areas, there could be greater aggregation of birds from anthropized environments that also use farms, such as spotless starlings, with other wild birds that do not frequent farms, thus resulting in a higher abundance of hosts to *Culex pipiens* s.l. In contrast, *Culex* species with feeding preferences for mammals, such as *Cx. theileri*, are slightly more abundant where large mammals are more likely to be encountered, on farms and in wild areas with abundant ungulates. The relatively similar general *Cx. theileri* abundance in the studied scenarios with the expected mixing of both *Cx. p. pipiens* and *Cx. p. molestus* further supports the relevance of the vector community in the emergence of WNV and other flaviviruses in these areas.

Undoubtedly, abiotic environmental conditions are highly influential on the biology, ecology and dynamics of mosquito populations (García‐Carrasco et al., 2023; Ruiz et al., 2010; Semenza et al., 2016), and this influence was also observed in our study. Both the local precipitation regime and the accumulated rainfall can affect mosquito population dynamics. In addition, precipitation may also influence other abiotic factors relevant to mosquito biological processes (Roiz et al., 2014). In wetlands, where the water supply generally comes from rainfall in distant areas, the local precipitation regime may not be a relevant predictor of the population dynamics of mosquitoes and their pathogens (Casades‐Martí, Cuadrado‐Matías, et al., 2023). However, in continental Mediterranean environments, with very severe hydric stress conditions in summer and in the absence of large water bodies (as in most of our study localities, except for some anthropized areas in S_6_ and S_7_), the only local water supply for mosquito oviposition and development sites (not counting the permanent water points artificially maintained by humans) is local accumulated rainfall. The positive effect of rainfall accumulation over a period of several months (2–7 months) corroborates the relevance of this predictor for the local abundance of WNV vectors. Temperature is an essential parameter in mosquito development, both in whether and how fast it occurs (Gardner et al., 2012), and favours vector competence of *Cx. pipiens* s.l. forms and their hybrids for flaviviruses (Deichmeister & Telang, 2011; Vogels et al., 2016). Previous studies identify the predominant role of environmental temperature in *Culex* spp. population dynamics (reviewed by Roiz et al., 2014), especially recent values prior to the time of capture (Roiz et al., 2014). Our findings are consistent with these observations and indicate the influence of recent temperature conditions on the abundance of these mosquitoes.

Our findings show that inner continental areas of peninsular Spain meet the required conditions for the presence of a diverse community of flavivirus vectors. Thus, and if we consider the positive effect of temperature on the abundance of these vectors, these areas could be even more favourable in the context of global warming; higher incidence of WNF and other flavivirus‐caused diseases would then be expected. Our study identified the environmental determinants of WNV vector population dynamics, which will allow applying our results to building new predictive models of vector abundance that could facilitate decision‐making for action against vectors and viruses. We corroborated previous results defining *Cx. pipiens* s.l. and *Cx. theileri* as the main vectors of WNV in the Spanish southern Plateau and observe that both species coexist in both farm and purely wild environments, probably favouring virus exchange between an enzootic bird–mosquito cycle and an epizootic mosquito–mammal cycle. The lack of effect of host abundance at the scale of the study may be related to the ad hoc selection of study areas or to the spatiotemporal scale of sampling, so it is advisable to focus the study of its influence in a different light. More comprehensive studies on the relevance of host community structure on WNV vector community structure and dynamics and WNV transmission risk are needed to more precisely estimate the risk of WNV infection for horses and humans.

## AUTHOR CONTRIBUTIONS

**Laia Casades‐Martí:** Conceptualization; investigation; writing – original draft; methodology; writing – review and editing; formal analysis; data curation. **Alfonso Peralbo‐Moreno:** Investigation; writing – review and editing; methodology; validation; software; data curation. **Sarah Delacour‐Estrella:** Investigation; writing – review and editing; validation; methodology. **Francisco Ruiz‐Fons:** Conceptualization; investigation; funding acquisition; writing – original draft; methodology; visualization; validation; writing – review and editing; project administration; formal analysis; data curation; supervision; resources.

## FUNDING INFORMATION

This work was supported by the Spanish Research Agency of the Ministry for the Science and Innovation (MCI) and the EU‐European Regional Development Fund (EU‐ERDF) through project E‐RTA2015‐0002‐C02. Laia Casades‐Martí and Alfonso Peralbo‐Moreno acknowledge the support of MCI, European Social Fund (EU‐ESF) and the University of Castilla‐La Mancha (UCLM) through contracts PEJ2018‐003155‐A and 2019‐PREDUCLM‐10932.

## CONFLICT OF INTEREST STATEMENT

The authors declare no conflicts of interest.

## ETHICS STATEMENT

The authors confirm that the ethical policies of the journal, as noted on the journal's author guidelines page, have been adhered to and the appropriate ethical review committee approval has been received. This study was conducted after the positive evaluation of the Animal Ethics & Experimentation Committee of the University of Castilla‐La Mancha and the approval by the competent authority of the regional government of Castilla‐La Mancha (Authorisation reference: 13‐2018).

## Supporting information


**Data S1:** Supplementary Information.

## Data Availability

Data are available at reference number doi: 10.5061/dryad.hhmgqnkrb.
