# Supplementary material for: Environmental determinants of West Nile virus vector abundance at the wildlife–livestock interface
Source: Med Vet Entomol. 2024 Nov 5;39(1):200–15. doi: 10.1111/mve.12774 (PMC11793132; doi:10.1111/mve.12774)

**Supplementary Table 1**. Set of models with ΔAICc < 2 selected for the average of models for the general *Culex* spp., the model for *Culex pipiens* and *Culex theileri*. The values refer to the estimated coefficients for each numerical predictor. Categorical variables selected by the models are denoted by a '+' symbol, those with no effect by 'ns', and those not included by a '-'..

| **Model set** | **Model**  **reference** | **ΔAICc/weight** | **Predictors** | | | | | |
| --- | --- | --- | --- | --- | --- | --- | --- | --- |
|  |  |  | **ung.ab** | **H1** | **igrad** | **AR.4_14** | **AT.0_1** | **AR.0_2** |
| *Culex* spp. | model 1 | 0.00/0.592 | 14.0000 | -3.261 | + | 1.899 | 2.768 | - |
|  | model 2 | 1.59/0.268 | ns | -2.705 | + | 1.890 | 2.782 | - |
| *Culex pipiens* | model 1 | 0.00/0.983 | - | -2.731 | + | 2.151 | 2.138 | - |
| *Culex theileri* | model 1 | 0.00/0.357 | - | -2.196 | ns | - | 5.571 | 0.09438 |
|  | model 2 | 0.52/0.276 | - | -2.134 | ns | - | 5.023 | ns |
|  | model 3 | 1.25/0.191 | - | -2.209 | + | - | 5.585 | 0.09744 |
|  | model 4 | 1.91/0.138 | - | -2.135 | + | - | 5.020 | ns |

**Supplementary Table 2**. Total number of captured mosquitoes per study site with specific indication of the number of captures for each of the 20 morphologically identified species. Whether each species was previously reported in Ciudad Real (CR) and Toledo (TO) provinces in previous studies is also displayed.

|  | **Number of mosquitoes captured** | | | | | | | | |  | **Previous presence** | |
| --- | --- | --- | --- | --- | --- | --- | --- | --- | --- | --- | --- | --- |
|  | **CIUDAD REAL (CR)** | | | | **TOLEDO (TO)** | | | | |  |  |  |
| **Species** | **S_1_** | **S_2_** | **S_3_** | **S_4_** | **S_5_** | **S_6_** | **S_7_** | **S_8_** | **S_9_** | **Total** | **CR** | **TO** |
| *Cx. pipiens* | 13 | 6 | 152 | 78 | 134 | 1864 | 408 | 43 | 453 | 3151 | yes | yes |
| *Cx. theileri* | 2 | 37 | 83 | 131 | 43 | 88 | 18 | 32 | 196 | 630 | yes | yes |
| *Cx. perexiguus* | 0 | 0 | 1 | 0 | 0 | 2 | 1 | 0 | 3 | 7 | yes | no |
| *Cx. modestus* | 0 | 0 | 0 | 0 | 0 | 1 | 0 | 0 | 0 | 1 | yes | no |
| *Cx. hortensis* | 2 | 0 | 0 | 0 | 1 | 1 | 1 | 0 | 0 | 5 | yes | yes |
| *Cx. laticinctus* | 0 | 0 | 0 | 0 | 0 | 1 | 0 | 0 | 0 | 1 | yes | no |
| ***Total Culex* spp.** | 17 | 43 | 236 | 209 | 178 | 1957 | 428 | 75 | 652 | 3795 | - | - |
| *An. maculipennis s.l.* | 1 | 0 | 2 | 17 | 1 | 5 | 2 | 0 | 10 | 38 | yes | yes |
| *An. claviger/petragnani* | 0 | 0 | 0 | 0 | 1 | 0 | 0 | 0 | 1 | 2 | yes | no |
| ***An.* spp.** | 2 | 1 | 3 | 23 | 2 | 8 | 3 | 3 | 14 | 59 | - | - |
| *Cs. longiareolata* | 3 | 6 | 1 | 3 | 3 | 60 | 30 | 0 | 11 | 117 | yes | yes |
| *Cs. annulata* | 0 | 0 | 0 | 0 | 0 | 4 | 1 | 1 | 5 | 11 | yes | yes |
| *Cs. subochrea* | 0 | 0 | 0 | 0 | 0 | 4 | 1 | 0 | 0 | 5 | yes | yes |
| *Cs. morsitans* | 0 | 0 | 0 | 0 | 0 | 0 | 2 | 2 | 0 | 4 | yes | no |
| ***Cs*. spp.** | 3 | 7 | 1 | 4 | 4 | 70 | 36 | 3 | 18 | 146 | - | - |
| *Ae. Vittatus* | 0 | 0 | 0 | 0 | 1 | 0 | 0 | 0 | 0 | 1 | yes | no |
| *Oc. caspius* | 0 | 0 | 0 | 0 | 0 | 3 | 1 | 0 | 15 | 19 | yes | no |
| *Oc. berlandi/pulcritarsis* | 1 | 2 | 3 | 1 | 9 | 5 | 1 | 12 | 10 | 44 | no | no |
| *Oc. geniculatus* | 0 | 1 | 0 | 1 | 0 | 0 | 0 | 2 | 2 | 6 | no | no |
| *Oc. detritus* | 0 | 0 | 0 | 0 | 0 | 0 | 0 | 0 | 1 | 1 | no | no |
| *Oc. rusticus/quasirusticus* | 0 | 0 | 0 | 0 | 0 | 0 | 0 | 1 | 0 | 1 | no | yes |
| *Oc. mariae/zammitii* | 0 | 0 | 0 | 0 | 0 | 2 | 0 | 0 | 0 | 2 | no | no |
| *Oc. pullatus* | 0 | 0 | 0 | 0 | 0 | 0 | 0 | 1 | 1 | 2 | no | no |
| ***Ae/Oc.* spp.** | 1 | 5 | 5 | 5 | 19 | 16 | 5 | 31 | 58 | 145 | - | - |
| *Or. pulcripalpis* | 0 | 0 | 0 | 0 | 0 | 0 | 0 | 0 | 1 | 1 | no | no |

**Supplementary Table 3**. Numbers of fortnight captures of *Culex* spp., *Cx. pipiens* and *Cx. theileri* throughout the different scenarios of wildlife-livestock interaction studied. NA: No survey.

|  | ***Cx. pipiens*** | | | | ***Cx. theileri*** | | | | ***Cx*. spp.** | | | |
| --- | --- | --- | --- | --- | --- | --- | --- | --- | --- | --- | --- | --- |
| **Sampling fortnight** | **A_1_** | **A_2_** | **A_3_** | **Total** | **A_1_** | **A_2_** | **A_3_** | **Total** | **A_1_** | **A_2_** | **A_3_** | **Total** |
| 1-15/05/2018 | 2 | 18 | 12 | 32 | 1 | 0 | 0 | 1 | 3 | 18 | 12 | 33 |
| 16-31/05/2018 | 27 | 84 | 51 | 162 | 14 | 11 | 2 | 27 | 41 | 95 | 53 | 189 |
| 1-15/06/2018 | 201 | 146 | 135 | 482 | 19 | 4 | 5 | 28 | 220 | 150 | 140 | 510 |
| 16-30/06/2018 | 36 | 239 | 38 | 313 | 6 | 7 | 22 | 35 | 42 | 246 | 60 | 348 |
| 1-15/07/2018 | 99 | 245 | 75 | 419 | 85 | 28 | 25 | 138 | 185 | 273 | 100 | 558 |
| 16-31/07/2018 | 147 | 221 | 39 | 407 | 31 | 24 | 39 | 94 | 179 | 245 | 78 | 502 |
| 1-15/08/2018 | 40 | 178 | 6 | 224 | 19 | 35 | 9 | 63 | 59 | 213 | 15 | 287 |
| 16-31/08/2018 | 29 | 191 | 119 | 339 | 13 | 2 | 9 | 24 | 42 | 194 | 128 | 364 |
| 1-15/09/2018 | 150 | 154 | 54 | 358 | 16 | 4 | 34 | 54 | 168 | 159 | 90 | 417 |
| 16-30/09/2018 | 25 | 67 | 48 | 140 | 6 | 15 | 12 | 33 | 32 | 82 | 60 | 174 |
| 1-15/10/2018 | 23 | 38 | 7 | 68 | 4 | 11 | 28 | 43 | 27 | 50 | 35 | 112 |
| 16-31/10/2018 | 4 | 3 | 1 | 8 | 1 | 0 | 0 | 1 | 6 | 3 | 1 | 10 |
| 1-15/11/2018 | 8 | 2 | 3 | 13 | 0 | 0 | 1 | 1 | 8 | 2 | 4 | 14 |
| 16-30/11/2018 | 0 | 0 | 0 | 0 | 0 | 0 | 0 | 0 | 0 | 0 | 0 | 0 |
| 1-15/12/2018 | 0 | 0 | 0 | 0 | 0 | 0 | 0 | 0 | 0 | 0 | 0 | 0 |
| 16-31/12/2018 | NA | NA | NA | NA | NA | NA | NA | NA | NA | NA | NA | NA |
| 1-15/01/2019 | NA | NA | NA | NA | NA | NA | NA | NA | NA | NA | NA | NA |
| 16-31/01/2019 | NA | NA | NA | NA | NA | NA | NA | NA | NA | NA | NA | NA |
| 1-15/02/2019 | NA | NA | NA | NA | NA | NA | NA | NA | NA | NA | NA | NA |
| 16-28/02/2019 | NA | NA | NA | NA | NA | NA | NA | NA | NA | NA | NA | NA |
| 1-15/03/2019 | NA | NA | NA | NA | NA | NA | NA | NA | NA | NA | NA | NA |
| 16-31/03/2019 | NA | NA | NA | NA | NA | NA | NA | NA | NA | NA | NA | NA |
| 1-15/04/2019 | 2 | 1 | 0 | 3 | 0 | 0 | 0 | 0 | 2 | 1 | 0 | 3 |
| 16-30/04/2019 | 0 | 12 | 1 | 13 | 1 | 1 | 0 | 2 | 1 | 14 | 1 | 16 |
| 1-15/05/2019 | 8 | 7 | 0 | 15 | 4 | 0 | 0 | 4 | 12 | 7 | 1 | 20 |
| 16-31/05/2019 | 2 | 0 | 0 | 2 | 0 | 3 | 0 | 3 | 2 | 4 | 0 | 6 |
| 1-15/06/2019 | 5 | 6 | 2 | 13 | 6 | 2 | 2 | 10 | 12 | 8 | 4 | 24 |
| 16-30/06/2019 | 29 | 44 | 5 | 78 | 9 | 1 | 4 | 14 | 38 | 45 | 9 | 92 |
| 1-15/07/2019 | 37 | 15 | 10 | 62 | 26 | 9 | 20 | 55 | 63 | 24 | 30 | 117 |
| Total | 874 | 1671 | 606 | 3151 | 261 | 157 | 212 | 630 | 1142 | 1833 | 821 | 3796 |

**Supplementary Table 4**. Indicator of mosquito host availability per site (S) and interaction gradient (A) calculated from the data obtained with the photo-trapping cameras as Σ (time of use x number of animals) / total camera placement time. The value for each site and interaction gradient has been multiplied by 10000 for easier understanding in the table.

| **Scenario** | **S_1_** | **S_2_** | **S_3_** | **S_4_** | **S_5_** | **S_6_** | **S_7_** | **S_8_** | **S_9_** | **TOTAL S_1_-S_9_** |
| --- | --- | --- | --- | --- | --- | --- | --- | --- | --- | --- |
| A_1_ | 0.00 | 561.32 | 58.00 | 0.00 | 52.36 | 0.00 | 0.00 | 189.30 | 0.00 | 860.98 |
| A_2_ | 7.92 | 0.00 | 125.61 | 39.82 | 37.39 | 15.12 | 0.00 | 22.34 | 0.00 | 248.20 |
| A_3_ | 6.66 | 0.83 | 38.17 | 50.09 | 1.74 | 6.99 | 295.34 | 2.12 | 33.83 | 385.68 |

**Supplementary Table 5**. Bird abundance indices per site and wildlife-livestock interaction scenario throughout bird taxonomic Order and Family. Within each taxonomic level, the highest abundance index of the three interaction scenarios is shown in bolded type letter case.

|  |  | **S_1_** | | | **S_2_** | | | **S_3_** | | | **S_4_** | | | **S_5_** | | | **S_1_-S_5_** | | |
| --- | --- | --- | --- | --- | --- | --- | --- | --- | --- | --- | --- | --- | --- | --- | --- | --- | --- | --- | --- |
| **Order** | **Family** | **A_1_** | **A_2_** | **A_3_** | **A_1_** | **A_2_** | **A_3_** | **A_1_** | **A_2_** | **A_3_** | **A_1_** | **A_2_** | **A_3_** | **A_1_** | **A_2_** | **A_3_** | **A_1_** | **A_2_** | **A_3_** |
| **Passeriformes** | *Passeridae* | **2.583** | 0.250 | 0.000 | **14.333** | 0.000 | 3.188 | 0.250 | 0.000 | **0.813** | **4.583** | 1.000 | 0.313 | **22.750** | 10.250 | 1.771 | **8.900** | 2.300 | 1.217 |
|  | *Hirundinidae* | **0.917** | 0.500 | 0.250 | 0.354 | 0.125 | **0.417** | 0.000 | 0.000 | 0.000 | **0.417** | 0.083 | 0.000 | 3.396 | **3.458** | 0.229 | **1.017** | 0.833 | 0.179 |
|  | *Lanidae* | **0.979** | 0.563 | 0.500 | 0.542 | 0.875 | **1.188** | 0.063 | 0.000 | **0.333** | **0.938** | 0.333 | 0.208 | 3.396 | **6.083** | 0.542 | 1.183 | **1.571** | 0.554 |
|  | *Alaudidae* | 0.271 | **0.875** | 0.083 | 0.646 | 0.146 | **0.896** | **0.896** | 0.479 | 0.063 | **0.688** | 0.208 | 0.625 | 0.000 | 0.000 | 0.000 | **0.500** | 0.342 | 0.333 |
|  | *Aegithalidae* | 0.250 | 0.063 | **0.583** | 0.000 | 0.000 | 0.000 | **0.250** | 0.000 | 0.125 | 0.000 | 0.083 | **0.417** | 0.000 | **3.042** | 0.125 | 0.100 | **0.638** | 0.250 |
|  | *Emberizidae* | **1.167** | 1.042 | 0.667 | 0.313 | 0.000 | **0.896** | **0.938** | 0.875 | 0.000 | **4.250** | 0.229 | 0.813 | **1.667** | 0.000 | 0.000 | **1.667** | 0.429 | 0.475 |
|  | *Motacillidae* | 0.000 | 0.000 | 0.000 | 0.000 | 0.000 | 0.000 | 0.000 | 0.000 | 0.000 | 0.000 | 0.000 | 0.000 | **0.458** | 0.000 | 0.083 | **0.092** | 0.000 | 0.017 |
|  | *Turdidae* | 0.167 | 0.125 | **0.271** | 0.000 | **0.958** | 0.000 | 0.292 | 0.125 | **0.604** | 0.000 | 0.000 | **0.229** | 0.083 | **0.625** | 0.063 | 0.108 | **0.367** | 0.233 |
|  | *Paridae* | 0.938 | **1.375** | 0.729 | 0.146 | **0.188** | 0.083 | **1.188** | 1.021 | **1.188** | 0.542 | 0.313 | **1.417** | 0.146 | **2.583** | 0.625 | 0.592 | **1.096** | 0.808 |
|  | *Sturnidae* | 1.375 | **1.792** | 0.063 | 1.813 | **3.583** | 0.000 | **3.229** | 0.708 | 0.000 | 0.188 | 0.438 | **1.729** | 83.313 | **137.500** | 2.083 | 17.983 | **28.804** | 0.775 |
|  | *Sylviidae* | 0.063 | **0.125** | **0.125** | 0.063 | **0.292** | 0.083 | 0.000 | **0.292** | 0.083 | 0.000 | 0.063 | **0.146** | 0.000 | 0.458 | **0.729** | 0.025 | **0.246** | 0.233 |
|  | *Corvidae* | **0.333** | 0.188 | 0.167 | 1.313 | **8.833** | 3.458 | 0.438 | 0.125 | **0.979** | 0.063 | 0.000 | **0.188** | 0.500 | **2.750** | 1.896 | 0.529 | **2.379** | 1.338 |
|  | *Cisticolidae* | 0.000 | 0.000 | 0.000 | 0.000 | 0.000 | 0.000 | **0.083** | 0.000 | 0.000 | 0.000 | 0.000 | 0.000 | 0.000 | 0.000 | **0.063** | **0.017** | 0.000 | 0.013 |
|  | *Fringilliidae* | 0.729 | **0.771** | 0.292 | 0.229 | 0.229 | **0.688** | 0.458 | 0.854 | **5.500** | 1.313 | 0.313 | **1.438** | **0.583** | 0.375 | 0.313 | 0.663 | 0.508 | **1.646** |
|  | *Sittidae* | 0.000 | 0.000 | **0.063** | 0.000 | 0.000 | 0.000 | 0.167 | 0.375 | **0.438** | **0.125** | 0.083 | 0.083 | 0.000 | 0.000 | 0.000 | 0.058 | 0.092 | **0.117** |
|  | *Muscicapidae* | 0.000 | **0.292** | 0.000 | 0.000 | 0.000 | 0.000 | 0.000 | **0.083** | **0.083** | 0.000 | **0.417** | 0.292 | **0.083** | 0.000 | **0.083** | 0.017 | **0.158** | 0.092 |
|  | *Cettidae* | **0.208** | 0.146 | 0.000 | 0.000 | 0.000 | 0.000 | 0.000 | 0.000 | 0.000 | 0.000 | 0.000 | 0.000 | 0.000 | 0.000 | **0.083** | **0.042** | 0.029 | 0.017 |
|  | *Oriolidae* | 0.000 | 0.083 | **0.229** | 0.000 | 0.000 | 0.000 | 0.229 | **0.542** | 0.000 | 0.000 | 0.000 | 0.000 | 0.000 | 0.000 | 0.000 | 0.046 | **0.125** | 0.046 |
|  | *Acrocephalidae* | 0.000 | **0.313** | 0.000 | 0.000 | 0.000 | 0.000 | 0.000 | 0.000 | 0.000 | 0.000 | 0.000 | 0.000 | **0.083** | 0.000 | 0.000 | 0.017 | **0.063** | 0.000 |
|  | *Phylloscopidae* | 0.000 | 0.000 | 0.000 | 0.000 | 0.000 | 0.000 | 0.000 | 0.000 | 0.000 | 0.000 | 0.000 | 0.000 | 0.000 | 0.000 | 0.000 | 0.000 | 0.000 | 0.000 |
|  | ***Subtotal*** | ***9.979*** | *8.500* | *4.021* | ***19.750*** | *15.229* | *10.896* | *8.479* | *5.479* | ***10.208*** | ***13.104*** | *3.563* | *7.896* | *116.458* | ***167.125*** | *8.688* | ***33.554*** | *39.979* | *8.342* |
| **Columbiformes** | *Columbidae* | **2.083** | 1.146 | 0.979 | 0.125 | **0.750** | 0.646 | **0.750** | 0.083 | 0.375 | 0.208 | 0.125 | **0.500** | **3.021** | 1.417 | 1.104 | **1.238** | 0.704 | 0.721 |
| **Charadriformes** | *Charadriidae* | 0.000 | **0.125** | 0.000 | 0.000 | 0.000 | 0.000 | 0.000 | 0.000 | 0.000 | 0.000 | 0.000 | 0.000 | 0.000 | 0.000 | 0.000 | 0.000 | **0.025** | 0.000 |
| **Cuculiformes** | *Cuculidae* | **0.063** | 0.000 | 0.000 | 0.000 | 0.000 | 0.000 | **0.063** | 0.000 | 0.000 | 0.000 | 0.000 | 0.000 | 0.000 | 0.000 | 0.000 | **0.025** | 0.000 | 0.000 |
| **Apodiformes** | *Apodidae* | 0.063 | 0.000 | **0.167** | 0.000 | **0.063** | 0.000 | 0.000 | 0.000 | 0.000 | 0.000 | 0.000 | 0.000 | 0.000 | 0.000 | 0.000 | 0.013 | 0.013 | **0.033** |
| **Bucerotiformes** | *Upupidae* | **0.292** | 0.208 | 0.083 | 0.000 | **0.563** | 0.083 | **0.063** | 0.000 | 0.000 | 0.000 | 0.000 | **0.125** | **0.646** | 0.000 | 0.000 | **0.200** | 0.154 | 0.058 |
| **Piciformes** | *Picidae* | 0.083 | **0.167** | 0.000 | 0.000 | **0.125** | 0.000 | 0.000 | **0.271** | 0.188 | 0.000 | 0.000 | 0.000 | 0.000 | 0.000 | 0.000 | 0.017 | **0.113** | 0.038 |
| **Accipitriformes** | *Accipitridae* | 0.000 | **0.313** | 0.000 | 0.000 | **0.083** | 0.000 | 0.000 | 0.000 | **0.063** | 0.000 | 0.000 | 0.000 | 0.063 | 0.000 | **0.083** | 0.013 | **0.079** | 0.029 |
| **Coraciiformes** | *Meropidae* | 0.000 | **5.250** | 0.313 | 0.000 | 0.000 | 0.000 | 0.000 | 0.000 | 0.000 | 0.375 | **0.604** | 0.000 | **0.146** | 0.083 | 0.125 | 0.104 | **1.188** | 0.088 |
|  | *Alcedinidae* | 0.000 | 0.000 | 0.000 | 0.000 | 0.000 | 0.000 | 0.000 | 0.000 | 0.000 | 0.000 | 0.000 | 0.000 | 0.000 | **1.375** | 0.000 | 0.000 | **0.275** | 0.000 |
|  | ***Subtotal*** | *0.000* | ***5.250*** | *0.313* | *0.000* | *0.000* | *0.000* | *0.000* | *0.000* | *0.000* | *0.375* | ***0.604*** | *0.000* | *0.146* | ***1.458*** | *0.125* | *0.104* | ***1.463*** | *0.088* |
| **Suliformes** | *Phalacrocoracidae* | 0.000 | 0.000 | 0.000 | 0.000 | 0.000 | 0.000 | 0.000 | 0.000 | 0.000 | 0.000 | 0.000 | 0.000 | **0.063** | 0.000 | 0.000 | **0.013** | 0.000 | 0.000 |
| **Pelecaniformes** | *Ardeidae* | 0.000 | 0.000 | 0.000 | 0.000 | 0.000 | 0.000 | 0.000 | 0.000 | 0.000 | 0.000 | 0.000 | **0.167** | 0.000 | 0.000 | 0.000 | 0.000 | 0.000 | **0.033** |
| **Ciconiidformes** | *Ciconiidae* | 0.000 | 0.000 | 0.000 | 0.000 | 0.000 | 0.000 | 0.000 | 0.000 | 0.000 | 0.000 | 0.000 | **0.063** | 0.000 | 0.000 | **0.146** | 0.000 | 0.000 | **0.042** |
| **Gruiformes** | *Ralliidae* | 0.000 | 0.000 | 0.000 | 0.000 | 0.000 | 0.000 | 0.000 | 0.000 | 0.000 | 0.000 | 0.000 | 0.000 | **0.333** | 0.000 | 0.000 | **0.067** | 0.000 | 0.000 |
|  | *Gruidae* | 0.000 | 0.000 | 0.000 | 0.000 | 0.000 | 0.000 | 0.000 | 0.000 | 0.000 | **2.021** | 0.000 | 0.438 | **2.250** | 0.000 | 0.000 | **0.854** | 0.000 | 0.088 |
|  | ***Subtotal*** | *0.000* | *0.000* | *0.000* | *0.000* | *0.000* | *0.000* | *0.000* | *0.000* | *0.000* | ***2.021*** | *0.000* | *0.438* | ***2.583*** | *0.000* | *0.000* | ***0.921*** | *0.000* | *0.088* |
| **Podicipediformes** | *Podicipediformes* | 0.000 | 0.000 | 0.000 | 0.000 | 0.000 | 0.000 | 0.000 | 0.000 | 0.000 | 0.000 | 0.000 | 0.000 | **0.229** | 0.000 | 0.000 | **0.046** | 0.000 | 0.000 |
| **Anseriformes** | *Anatidae* | 0.000 | 0.000 | 0.000 | 0.000 | 0.000 | 0.000 | **0.125** | 0.000 | 0.000 | 0.000 | 0.000 | 0.000 | 0.000 | 0.000 | 0.000 | **0.025** | 0.000 | 0.000 |
| **Galliformes** | *Phasianidae* | 0.000 | 0.000 | **0.083** | 0.000 | 0.000 | 0.083 | **0.063** | 0.000 | 0.000 | 0.000 | 0.000 | 0.000 | 0.000 | 0.000 | 0.000 | 0.013 | 0.000 | **0.033** |
| ***TOTAL*** | | ***12.563*** | ***15.708*** | ***5.646*** | ***19.875*** | ***16.813*** | ***11.708*** | ***9.542*** | ***5.833*** | ***10.833*** | ***15.708*** | ***4.292*** | ***9.188*** | ***123.208*** | ***170.000*** | ***10.146*** | ***36.179*** | ***42.529*** | ***9.504*** |

**Supplementary Figure 1**. Mosquito trapping station including a CDC-type miniature white light trap at 1.8 m from the soil, a BG sentinel trap on soil and the CO_2_ deliver device on top of the traps (2.2m height).


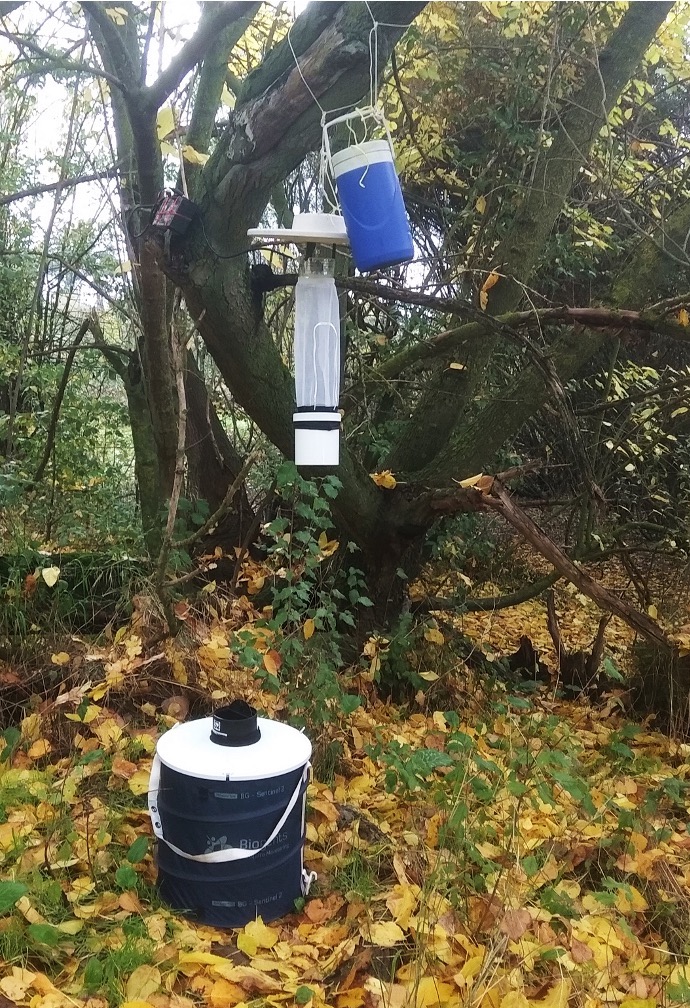


**Supplementary Figure 2**. Camera trap images showing the use of the mosquito trapping space by wild ungulates (A: fallow deer (*Dama dama*) buck; B: red deer (*Cervus elaphus*) stag; C: Eurasian wild boar (*Sus scrofa*); and D: roe deer (*Capreolus capreolus*) doe).


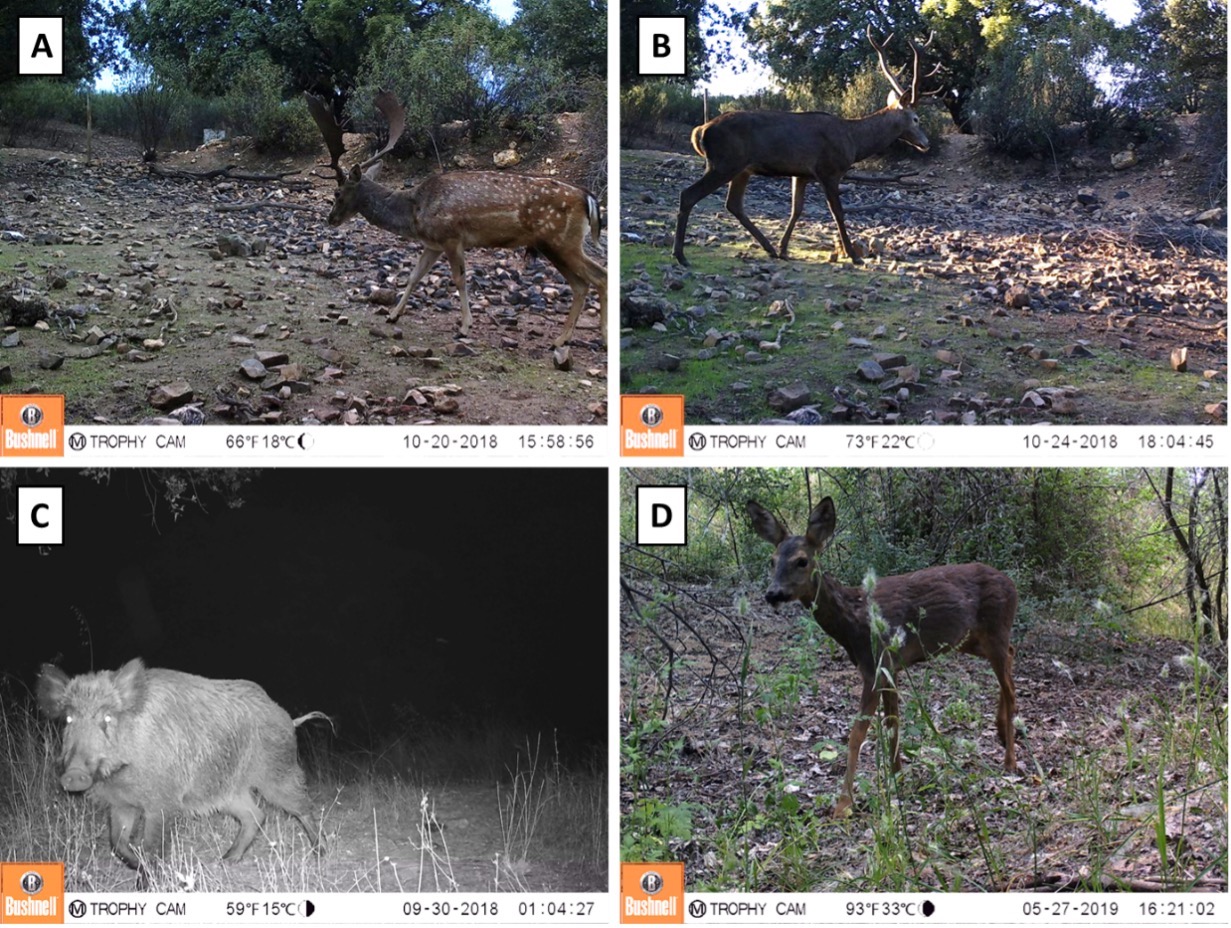


**Supplementary Figure 3.** Fortnight evolution of *Culex* spp. abundance (black line) in relation to the temporal variation of time lagged values of temperature (red line) and accumulated precipitation (blue line) per study site.


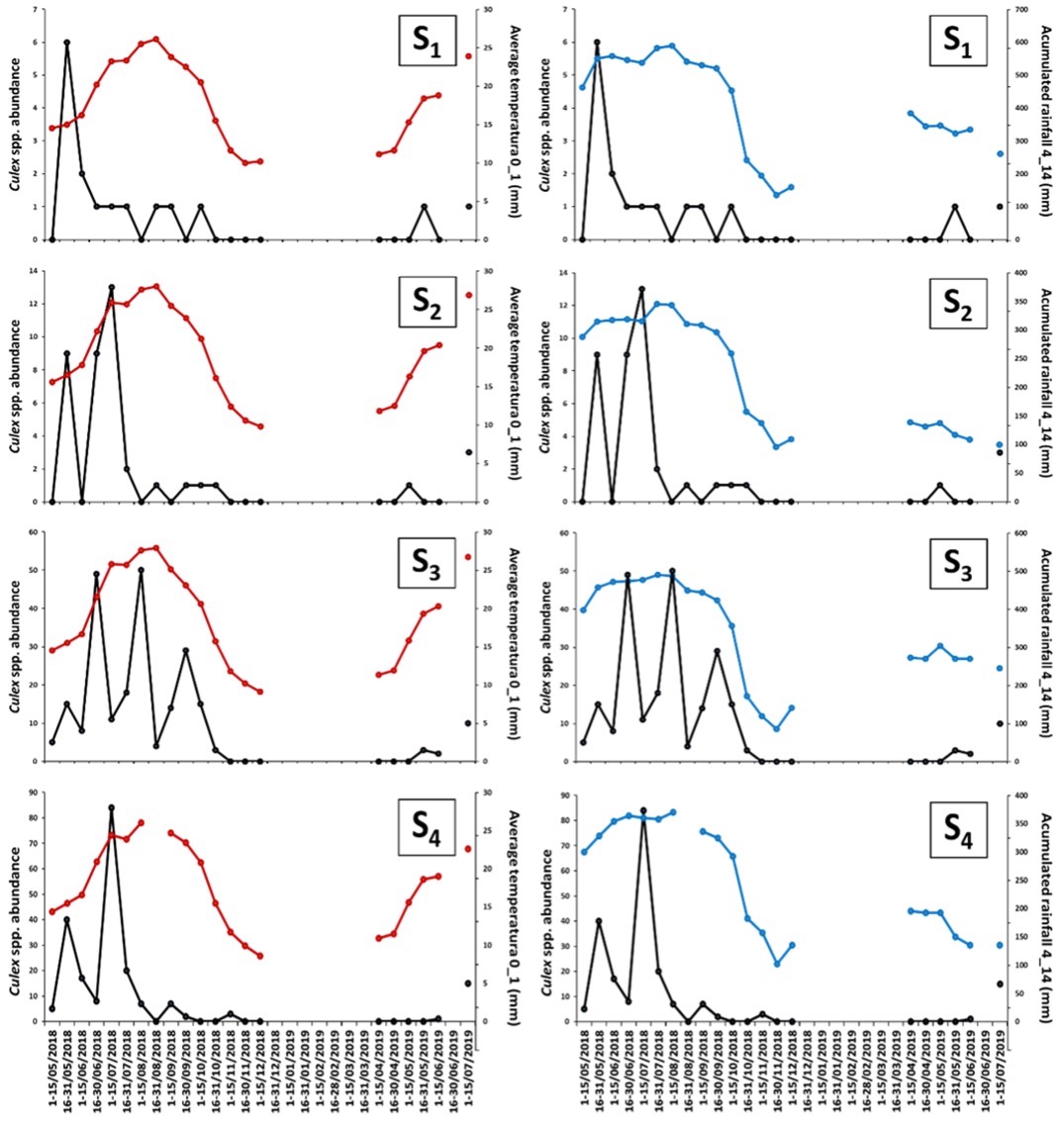


**Supplementary Figure 3 (continued).**


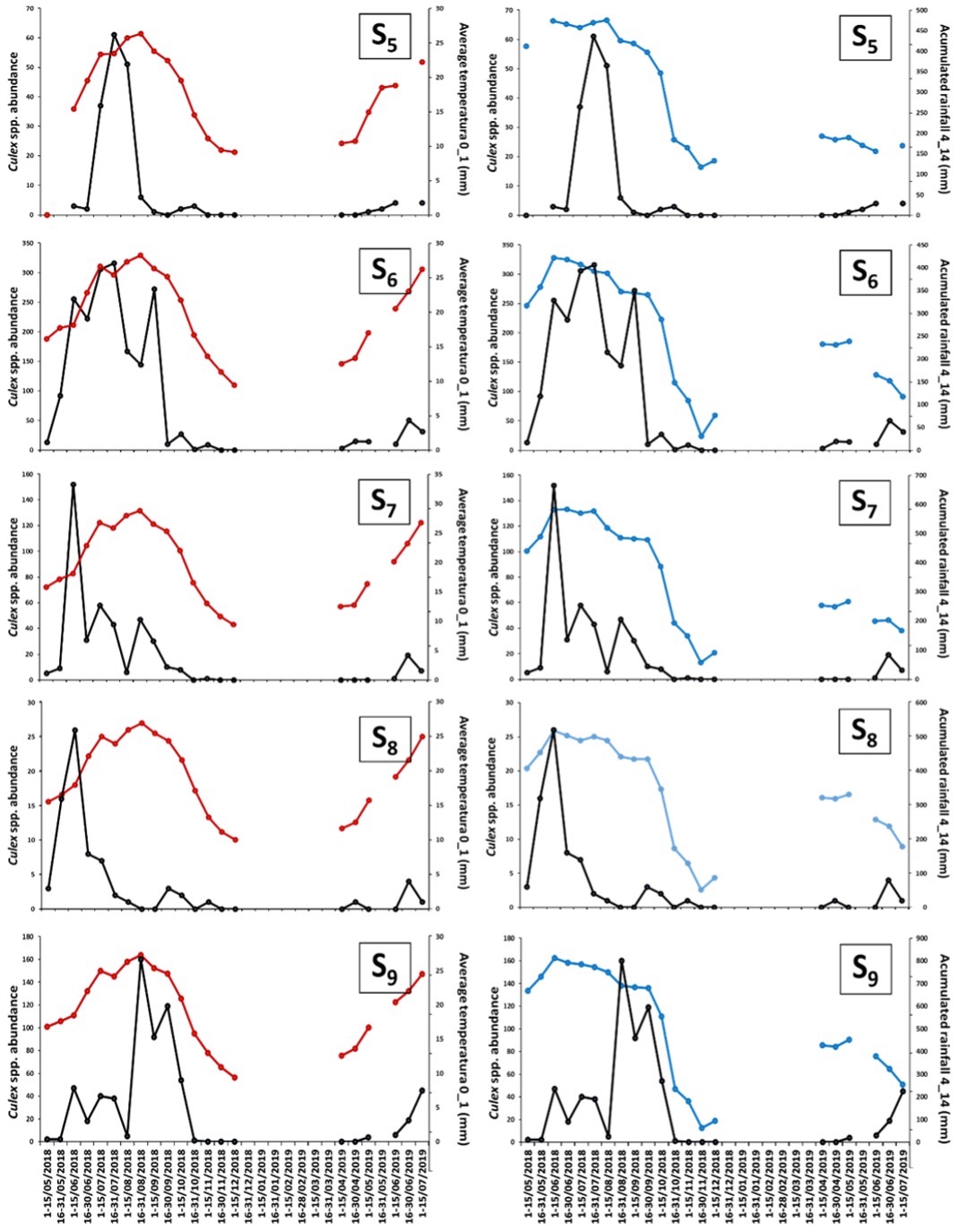

Supplement: Supplementary file 1 — Data S1: Supplementary Information. [file MVE-39-200-s001.docx]
